# A Sensory-Driven Trade-Off between Coordinated Motion in Social Prey and a Predator’s Visual Confusion

**DOI:** 10.1371/journal.pcbi.1004708

**Published:** 2016-02-25

**Authors:** Bertrand H. Lemasson, Colby J. Tanner, Eric Dimperio

**Affiliations:** 1 Environmental Laboratory, U.S. Army Engineer Research & Development Center (ERDC), Santa Barbara, California, United States of America; 2 Department of Ecology, Université de Lausanne, Switzerland; Princeton University, UNITED STATES

## Abstract

Social animals are capable of enhancing their awareness by paying attention to their neighbors, and prey found in groups can also confuse their predators. Both sides of these sensory benefits have long been appreciated, yet less is known of how the perception of events from the perspectives of both prey and predator can interact to influence their encounters. Here we examined how a visual sensory mechanism impacts the collective motion of prey and, subsequently, how their resulting movements influenced predator confusion and capture ability. We presented virtual prey to human players in a targeting game and measured the speed and accuracy with which participants caught designated prey. As prey paid more attention to neighbor movements their collective coordination increased, yet increases in prey coordination were positively associated with increases in the speed and accuracy of attacks. However, while attack speed was unaffected by the initial state of the prey, accuracy dropped significantly if the prey were already organized at the start of the attack, rather than in the process of self-organizing. By repeating attack scenarios and masking the targeted prey’s neighbors we were able to visually isolate them and conclusively demonstrate how visual confusion impacted capture ability. Delays in capture caused by decreased coordination amongst the prey depended upon the collection motion of neighboring prey, while it was primarily the motion of the targets themselves that determined capture accuracy. Interestingly, while a complete loss of coordination in the prey (e.g., a flash expansion) caused the greatest delay in capture, such behavior had little effect on capture accuracy. Lastly, while increases in collective coordination in prey enhanced personal risk, traveling in coordinated groups was still better than appearing alone. These findings demonstrate a trade-off between the sensory mechanisms that can enhance the collective properties that emerge in social animals and the individual group member’s predation risk during an attack.

## Author Summary

Many social species coordinate their movements as they travel together, which is generally considered to be an adaptive behavior predominantly mediated by vision. Collective coordination lowers the individual’s chances of becoming separated from the group and can reduce the time spent in risky areas. More broadly, information is believed to spread more rapidly among individuals as group level order increases. Yet, little attention has been payed to how collective order impacts a predator’s perception of their prey during an attack. Motion detection improves with the coherency of visual stimuli, which suggests that the sensory mechanisms that promote coordinated motion in prey may also improve predator perception and, consequently, attack success. Here we explore this conundrum using an interactive game with humans acting as surrogate predators attacking collections of virtual prey. By isolating visually mediated behaviors in both prey and predator we demonstrate the potential for a sensory-driven trade-off to arise between a group’s collective order and the individual’s risk of being captured.

## Introduction

Organisms are constantly challenged by the need to effectively extract and process pertinent information from uncertain and often dangerous environments. Many species manage to reduce their uncertainty through social information. When animals gather together and coordinate their activities the members can enhance their own perception through collective vigilance, which increases the speed and accuracy of individual decisions and reduces predation risk [1–3]. Predators can also be cognitively challenged when presented with multiple targets (the confusion effect), thereby lowering their ability to process information and, consequently, further reducing a prey’s risk of being killed. It is clear that social interactions can influence perception in both social prey and their predators, yet we still know little of how these opposing processes can interact to influence predator-prey encounters.

Collective vigilance is an important anti-predatory benefit of social life that depends upon how group members pay attention to one another. Pulliam’s original mathematical argument (1973) assumed that once one member of the group is aware of a threat that the information is immediately public. In reality information needs to propagate across members and transmission rate is limited by individual perception, predominantly through auditory and/or visual monitoring [4–6]. For animal groups on the move, the quality of their social communications is generally measured by how well they can coordinate their actions. The costs or benefits of such collective coordination are likely to be particularly acute during transition phases, as when groups initially coalesce and respond to a perturbation. Unfortunately, the mechanisms for how animals integrate social cues and coordinate their activities remain elusive, but data indicate that individuals often rely on vision for rapid responses to changes in their neighbors’ movements (bees, [7]; fish, [8, 9]; humans, [10]). Recent theory suggests that selective attention to motion cues can expedite the sensory integration process in animal groups, substantially enhancing the speed and accuracy with which social animals coordinate their actions [11].

Motion is a critical factor in predation, although a prey’s speed and turning behavior can have varying effects on a predator’s capture ability. An animal’s speed can initially attract a predator’s attention, thereby increasing the risk of detection and attack [12, 13], yet once driven to action animals will display impressive escape speeds to avoid capture [14, 15]. Increased turning rates are believed to reduce predation risk by disrupting a predator’s ability to visually track their quarry and predict an intercept course [16], although evidence for this is mixed [17–19]. Prey turning behavior also appears to be less influential in predator attacks than prey speed and the effect of either of these velocity components can be reversed when a predator must consider multiple targets at once [20, 21].

Prey found in groups can further reduce predator capture success through the confusion effect, although when and how group-level movements contribute to this process remains unclear. Multiple competing stimuli divide a predator’s attention, overwhelming its ability to select any one prey, thereby reducing its capture ability [2, 4, 22]. The confusion effect is generally associated with changes in either the number or density of prey [23–26], yet confusion can begin with a pair of prey [20] and density effects are context dependent [27–29]. Jones and her colleagues [17] demonstrated that randomly moving particles were harder for human subjects to capture when these asocial particles moved more erratically and increased in number. What remains to be tested is how even simple social feedbacks will impact a prey’s risk during an attack and, more importantly, whether any predator confusion derives from the targeted individual’s socially driven movements, visual distractions caused by neighbors, or both.

How coordinated social movements influence predation risk remain largely unexplored. Social coordination statistically reduces movement variability, which should be detrimental for moving prey since predators can be very efficient at visually tracking targets [18, 19]. Additionally, random-dot assays demonstrate that visual perception of motion improves with the coherency, or coordination, of collective stimuli (fish, [30]; birds, [31]; primates, [32]; humans, [31]). If coordinated motion reduces movement variability and improves capture ability in visual species, then we should expect a trade-off between enhancing information transmission among group members and reducing an individual’s risk of being captured during an attack.

In this study we looked for evidence of a trade-off between social coordination and predation risk by testing the hypothesis that increases in prey social coordination can reduce visual confusion in a predator. Specifically, we explored how socially influenced directional feedbacks in social prey impact predator capture ability. To test our hypothesis we used a visual-based social model to generate virtual prey in a targeting game in which human participants acted as surrogate predators. The prey model incorporates motion-guided attention into a self-organizing process, whereby individuals react to the movements of their neighbors based on those visual cues that exceed a sensory threshold [11]. We then projected our virtual prey onto a computer screen and participants were tasked with capturing a targeted prey within a group using a mouse. Our virtual prey could only perceive one another and could not respond directly to the attack, which avoids confounding responses based on personal *vs*. social information in the prey and reflects scenarios in which most of the group depends on social information when responding to disturbances [33, 34]. Capture ability was measured by recording player capture latency (time until clicking on a prey item) and accuracy (distance from the prey item when clicked).

We first determined how the prey’s visual sensory thresholds, their speed, and initial state influenced capture ability. Tuning the preys’ sensory thresholds allows us to explore links between prey visual perception and its impacts on a predator’s visual performance. To address the potential for any ‘behavioral oddity’ that may impact risk profiles we then independently varied the target’s sensory thresholds from those of the remaining group members. In nature risk profiles may also vary based on the initial state of the prey at the start of an attack. To address this we controlled the initial speed of the targeted prey and the overall state of the group (disordered, ordered), which enabled us to explore very different ecological conditions. Lastly, we used a novel means of testing for visual confusion by repeating the above scenarios, but veiling the target’s neighbors. By adopting this approach we controlled for the presence of visual distractions created by neighboring prey, while retaining their collective influence on the target’s movements.

## Materials and Methods

### Ethics statement

Pilot trials were conducted at various locations in the United States, including government buildings and scientific society meetings, while the experimental data presented here were collected from 30 consenting adult subjects from l’Université de Lausanne, Switzerland. U.S. trials were approved by Portland State’s Institutional Review Board (#122144) and ethical approval at l’Université de Lausanne was provided by the Secrétariat de la Commission cantonale (VD) d’éthique de la recherche sur l’être humain. Experimental protocols were identical across locations.

### Model

We simulated groups of prey by modifying a particle model in which social coordination stems from how individuals visually perceive and react to the movement decisions of their neighbors [11]. The model’s basic structure builds upon earlier works to study how basic cognitive mechanisms can influence animal movement mechanics in dynamic environments [35]. Here, the value of each neighbor’s movements is weighted according to an observer’s visual perspective:

$$v_{j,i}=\omega_{j,i}\cdot {\hat{v}}_{j,i},$$ (1)

where ${\hat{v}}_{j,i}$ is neighbor *j*’s current direction relative to subject *i* and *ω*_*j*,*i*_ scales the influence of that vector based on the observer’s perception (see section S1.1 in the S1 Text for further details). Based on what individuals can see we then assume that all of this information is filtered, so that each group member selectively responds to a set of **N**_*i*_ neighbors whose motion cues are strong enough to stand out in their field of view:

$$j\in N_{i}\;\text{if}\;\frac{\omega_{j,i}}{\bar{\omega }}\geq m$$ (2)

Here $\bar{\omega }$ is the mean speed of the individual’s optic flow and parameter *m* is a motion threshold that tunes how sensitive individuals are to the changes in the relative speeds of their neighbors. Eq 2 represents a generalization of the signal-to-noise property of Weber’s Law, in which the perception of a stimulus is some constant proportion of the background. Individuals then attempt to adjust their velocity according to this social information, $v_{i}^{s}$, which is represented by the average degree of relative motion that has grabbed their attention:

$$v_{i}^{s}=v_{i}+{\left\langle \omega_{j,i}\cdot {\hat{v}}_{j,i}\right\rangle }_{N_{i}}$$ (3)

**Fig 1:**
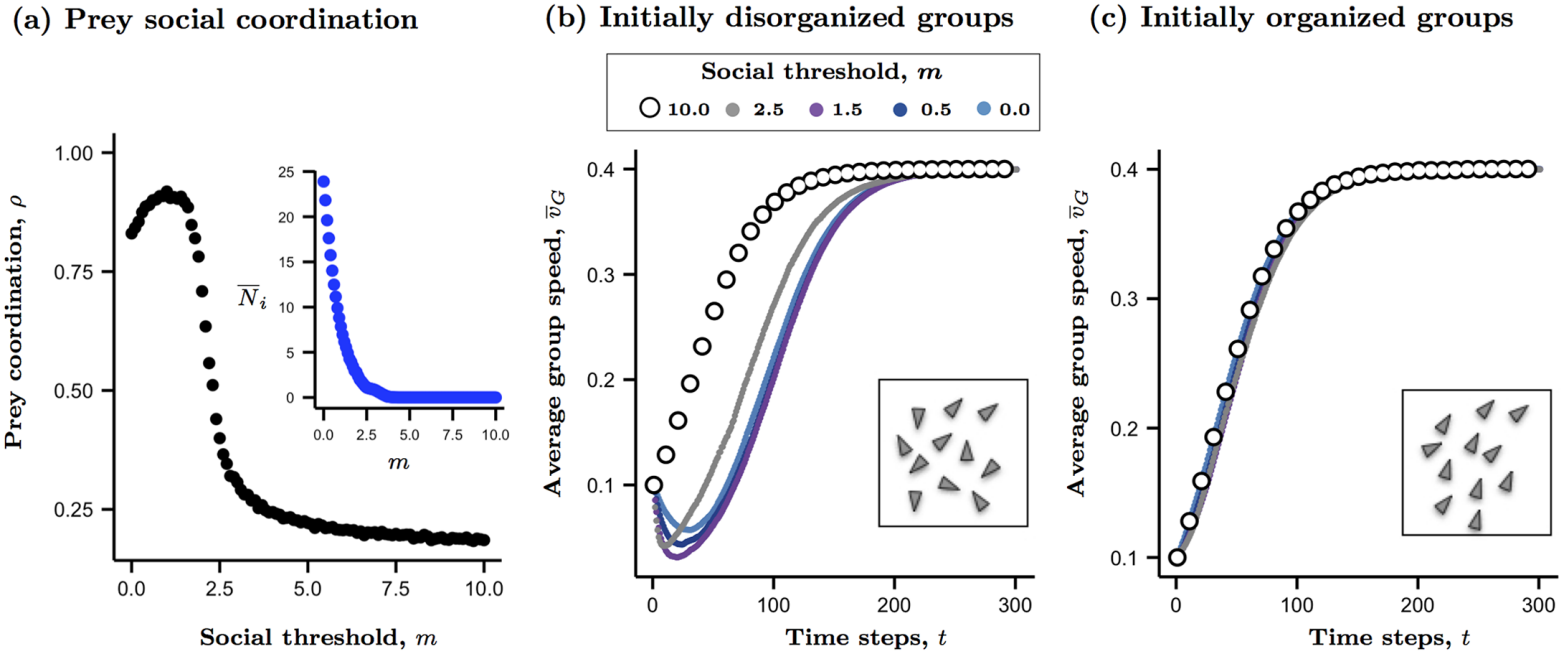
The influence of individual social thresholds and initial group organization on attention levels, collective coordination, and average individual speed. As the individual’s social threshold increases (*m*), overall coordination in the group begins to rise then decays rapidly (a) because individual’s are less likely to pay attention to their neighbors (a, inset). Over time, the group’s average speed is a function of both the individual-level interactions and the initial conditions (b, c). Increases in social thresholds range from asocial behavior (random walk; black open circles) to social interactions of varying degrees (colored points). Insets in (b) and (c) illustrate the initial orientations of the prey. Increased social attention is reflected by a larger number of influential neighbors, *N_i_* = |**N**_*i*_| (Eq 4). When groups are initially disorganized (b) a lag in the average individual’s departure speed emerges as a consequence of the time needed to come to a directional consensus. When groups are already organized their social interactions have little feedback on collective speed (c). Model parameters: group size = 25, *m*: {0, 1.5, 2.5, 10.0}, v* = 0.44. Data represent mean values of 1,000 replicates per setting. Additional parameters are provided in S1 Table.

The individual’s motion threshold therefore acts as a sensory mechanism that dictates its level of social interaction, and therefore, the overall degree of coordination that emerges. For brevity we will simply refer to *m* as the prey’s social threshold from here on. Groups composed of individuals with low social thresholds display highly coordinated motion (Fig 1a), which decays rapidly as individual thresholds increase because members pay less attention to one another’s actions (Fig 1a inset). Note that when individual thresholds are essentially unconstrained (*m* = 0) the resulting degree of social coordination initially improves before decaying as *m* continues to increase. This pattern aligns with the hypothesis that an unconstrained sensory range within a group can actually reduce directional coordination, since distant neighbors are less likely to behave in a similar fashion [2].

Each individual’s future step is then an attempt to adapt to its current social settings, whereby individuals effectively scale their reaction to any social directional cues according to their own internal state:

$$v_{i}\left(t+\Delta t\right)=v_{i}^{s}\;\left[1-\gamma (v_{i}^{s})\right]\;{\hat{v}}_{i}^{s}\;\left(t\right)$$ (4)

Here $v_{i}^{s}$ and ${\hat{v}}_{i}^{s}$ represent the speed and directional components of an individual’s socially adjusted velocity, $v_{i}^{s}$. Function *γ*() is a biological extension of the dampening term used in the application of Langevin dynamics in social force models [36]. For our purposes we’ve generalized this physical term to a cost function to reflect the tendency for animals to modify their speeds in response to both energetic and ecological stressors (see S1 Fig and section S1.2 in the S1 Text). Individuals track their current speed relative to an expected optimum (v*), which reflects a population-level behavior (average travel speed) that stems from individual-level capabilities. When traveling as a group such speed control not only ensures that all members travel at the same speed, but effectively causes group members to accelerate when they fall behind and decelerate when they pull away from their neighbors (*v*_*i*_ < v* *vs*. *v*_*i*_ > v*, respectively). Recent empirical work has demonstrated that such speed dynamics are an important component in collective motion and information transfer [37–39].

Individuals then update their positions discretely based on their desired velocity:

$$x_{i}\left(t+\Delta t\right)=x_{i}\left(t\right)+\left[v_{i}\left(t+\Delta t\right)+U\left(v_{i},\eta \right)\right]\Delta t$$ (5)

Function *U* adds stochasticity to the process by independently adjusting speed and direction by a magnitude of *η*, where *η* ∈ [0, 1]. Speed varies by ± *η* ⋅ *v*(*t*), while the heading $\hat{v}\left(t+\Delta t\right)$ is rotated by an angle between [−*ηπ*/2, *ηπ*/2]. Prey turning rate was limited to (*π*/2)/*Δt*. Time is given by each simulation step *t* and distance units are generic, being normalized to particle diameter (*D* = 2*r*). See S1 Table for additional details).

Directional preferences in the model are predominantly driven by the degree to which individuals respond to changes in the relative velocities of their neighbors (Eq 3). By including an ecological feedback to control individual velocity (Eq 4) we can generate new dynamics to explore an important consequence of social behavior, namely, the interaction between the individual’s social threshold (here, sensitivity to neighbor motion), their resulting velocity, and, finally, the emergent degree of social coordination. Consider a group of foragers departing an area by moving off at random. With little social feedbacks in their directional choices, each individual may show only minor deviations in direction (given by *U* in Eq 5) as they accelerate to their expected travel speed (Fig 1b, open circles). Such asocial behavior can also occur during predation events and is typified by the so-called flash expansion effect observed in fish schools under attack [14]. However, when individuals mimic the decisions of their neighbors this behavior causes a directional feedback that propagates across the group and delays the average individual’s departure speed (Fig 1b). Here the extent of this departure lag is driven by each individual’s social threshold, *m* in Eq 2. As *m* drops, individuals take longer to achieve a directional consensus as they pool information across an increasing number of neighbors and forward momentum is delayed. While the final speed achieved is unaffected by the underlying social interactions, the initial lag in departure speed during self-organization may increase a targeted individual’s risk of being captured by a predator. Given the feedback between social coordination and group speed, the initial state of the group should also affect predation risk. If prey are already aligned at the start of a predator’s attack, such as a group of fish aligned in a current, then any socially driven effects on the average individual’s speed due to turning behaviors should be dampened because directional differences are either ignored or marginalized (Fig 1c; Eq 5).

### Game

The use of interactive games that rely on virtual prey and or surrogate predators has steadily grown as a practical means of minimizing unnecessary harm, or controlling for confounding factors like the state of the individuals (fish, [21]; birds, [40]; humans, [17, 25, 28, 41]). Here we designed an interactive game to test how a targeted prey’s initial escape speed, social threshold, and the initial organization of its group members could affect a predator’s capture ability. The game began by displaying instructions outlining the goal and the rules. Participants were asked to sit behind a portable screen and wear earplugs to reduce both visual and auditory distractions. Prey appeared in the center of the screen either alone or in a group of 25 and a player’s goal was to click on the designated target before it escaped by moving off-screen. The target was highlighted in red for 250 *ms* before turning the same color as the remaining prey (for an example see S1 Video). Players had to pass a practice round to become familiar with the mouse and screen settings, and could not proceed onto the data collection phase unless they captured 4/5 practice targets. If a target moved off-screen before a player could capture it the session was counted as a miss and terminated. We recorded the movements of the mouse and all virtual prey every 20 *ms*, and these data were used to calculate player capture latency and accuracy. Failure to record a mouse click during a trial was flagged and the activity of the virtual prey and player’s mouse movements were reviewed. There were 13 such trials, which together constituted less than 1% of the data collected and we corrected for any spurious effects prior to our analyses (See section S2.1 in the S1 Text for details).

Each player completed 144 trials of different parameter combinations and on average the game took 10 minutes to complete (including training), with each trial lasting between 240–5,767 *ms* (median time = 1,141 *ms*, or 1.14 *s*). Games were run on a 15.4” MacBook Pro under 1440 x 900 resolution (110 *pixels* per *inch*). At the start of each trial the average prey member accelerated up to a mean speed of 0.400 *D*/*t*, where *D* was equivalent to 1 pixel and *t* is a simulation step. Prey speed was limited to a minimum of 0.1 *D*/*t* because pilot trials indicated that slower speeds were too easy for participants. Animations were rendered at 50 frames per second, yielding particle speeds ranging from 0.12 (min.), 0.46 (mean) and 1.15 (max.) *cm*/*s*. All prey particles were rendered to appear approximately 0.95 *cm* long.

We organized our procedures into three distinct sessions (I–III). The conditions and parameter settings for each of these sessions were determined a priori based on earlier pilot trials conducted during the summer of 2013. As such, all parameter combinations were presented to players in a randomized, full factorial design rather than incrementally in distinct experiments (Table 1). The graphical interface of the game was coded in the Java-based Processing platform (version 1.5.1) and all data processing and statistical analyses were done in R (version 3.0.2) [42].

**Table 1:** Game response variables, factors, and continuous predictor variables.

**Response variables**	**Description**	**Range**	**Units**
*P*_*A*_	Player capture accuracy	[0.0, 1.0]	-
*P*_*L*_	Player capture latency	[6.2, 8.4]	log(*ms*)
**Factors**	**Description**	**Levels**	**Units**
*m*_*T*_	Target motion threshold	[0.5, 1.5, 2.5, 10]	-
*m*_*N*_	Neighbors motion thresholds	[0.5, 1.5, 2.5, 10]	-
*v*_*e*_	Target initial escape speed	[1, 10]	*D* ⋅ *t*^−1^
*ρ*_0_	Initial group organization	0.27, 0.9	-
veiled	Neighbors veiled	Yes, No	-
**Continuous variables**	**Description**	**Range**	**Units**
*v*_*T*_	Target speed	[0.1, 1.5]	*D* ⋅ *t*^−1^
*tor*	Target path tortuosity	[0.0, 1.0]	-
*vpa*	Target Voronoi polygon area	[3.6, 9879.0]	pixels^2^
*v*_*G*_	Group speed	[0.1, 0.7]	*D* ⋅ *t*^−1^
*ρ*	Group order	[0.0, 1.0]	-
${\bar{d}}_{1}$	Mean nearest neighbor distance	[0.0, 44.4]	*D*

#### Session I: Target speed, social thresholds, and collective state

We manipulated three primary factors: a targeted prey’s initial escape speed (*v*_*e*_), prey social threshold (*m*; Eq 3), and the initial degree to which the prey were organized, or aligned, *ρ*_0_(Table 1). During an attack by a predator most social animals will either be surprised, or incapable of directly detecting the threat, and, in either case, members will primarily be responding to the behaviors of their neighbors (e.g., the Many-eyes hypothesis; [2]). To reflect these conditions we adopted a minimalist approach whereby we assumed that either none of the prey were aware of the attack and all moved off at the same speed (*v*_*e*_ = 1), or only the targeted prey had spotted the predator and attempted to burst away (*v*_*e*_ = 10). These alternative conditions were implemented by scaling the targeted prey’s speed within the first 5 frames of a given trial as *v*_*T*_(*t*) = *v*_*e*_ ⋅ *v*_*T*_(*t* − *Δt*), where *v*_*T*_ specifies the targeted prey’s speed. For those scenarios in which prey were initially disorganized, individuals were assigned random orientations, ${\hat{v}}_{i}\in \left[0,2\pi \right]$. Under organized starting conditions we randomly assigned a global direction to all group members and then wobbled each individual’s heading by ± 0.175 radians (10°) to retain some degree of inherent individual variability.

#### Session II: Sensory heterogeneity

Here we explored how differences in individual sensory thresholds among prey can impact a predator’s capture ability by independently varying the target’s social threshold (*m*_*T*_) relative to those of its remaining group members (*m*_*G*_). Target and group member social thresholds varied equally in a full factorial combination (*m*_*T*_ x *m*_*G*_, respectively; Table 1). For ecological relevance and practical purposes (time limitations) we limited these combination of trials to instances when a target was initially startled (*v*_*e*_ = 10). Under natural conditions animals within a group are more likely to differ from their neighbors if they are intentionally adopting a different strategy to evade capture than if they are unaware of any threat.

#### Session III: Visual confusion effect

To determine if the perceived motions of a targeted prey’s group members confused players, we repeated the above protocol of session II, but introduced a veiled condition, whereby the targeted prey’s fellow group members were not rendered on-screen. For this we stored the random seed used to generate each simulation in which all group members were visible and used the same seed to generate a veiled replicate trial. The order in which a player experienced visible/veiled conditions was randomized in the final design. Prey in the model therefore repeated the exact movements across visible/veiled pairings, which allowed us to isolate the visual effects of individual *vs*. collective motion on predator capture ability.

### Statistics

Players were presented with two replicates of each parameter combination, and we used linear mixed effects models (LMM) to determine if our primary factors (*v*_*e*_, *m*_{*T*,*G*}_, *ρ*_0_, and veiled) significantly affected the latency (*P*_*L*_) and accuracy (*P*_*A*_) with which players captured their targets. Latency represents the time (*ms*) taken in a trial to click on the target. Accuracy represents the normalized distance between the mouse cursor and the target’s center of mass when the player attempted to capture the target by clicking on or near it. For analyses, latency was log transformed, and accuracy was transformed so that *P*_*A*_ = 1 − (log(*d*_*T*_ + 1)/max(log(*d*_*T*_ + 1))), where *d*_*T*_ was the distance from the mouse to the target in pixels [29]. All *d*_*T*_ values were reviewed for edge effects and adjusted if necessary to avoid spurious *P*_*L*_ or *P*_*A*_ values (See S2 Fig and section S2.1 in the S1 Text). *P*_*A*_ values range from 0–1 (furthest to closest recorded values). The social threshold values, *m*, were also transformed for the LMM analyses as L(m)=log(m+1). Variance within player was included as a random effect, and all initial models included the predictor variables relevant to each of the above experimental conditions and all possible interactions. We generated final models by removing insignificant (*P* > 0.05) terms, beginning with highest-order interactions and working to main effects, using analysis of deviance tests on nested models when needed [43].

### Kinetic analyses

To better understand why our factors influenced player capture ability we conducted a secondary set of analyses to explore how the underlying kinetics of prey motion affected predator latency and accuracy. Kinetically derived properties (i.e., those derived from the prey’s movements) were partitioned into local and global functional groups to explore the potential for differences between these scales. Local metrics included spacing around the target, along with its speed and turning behavior. Correspondingly, global metrics included average nearest neighbor distance, group speed and collective order (Table 1). Local spacing around each target was measured using its Voronoi polygon area, *vpa*, which was calculated using the **sp** and **deldir** libraries in R [44, 45]. A target’s *vpa* represents its personal space, whereby every point within the polygon is closer to the target than to any of its nearby neighbors. This metric effectively measures a prey’s risk of being captured by a predator [46]. Global spacing was measured using the average nearest neighbor distance across all particles [29] and group speed was simply the mean speed of all prey. A target’s turning behavior was measured by the tortuosity of its movements, *tor*. Tortuosity is a dimensionless metric that is inversely related to how much an object’s movements deviate from a straight line [47]:

$$tor=1-\left(\frac{|x_{\text{final}}-x_{0}|}{\sum l_{t}}\right),$$ (6)

where the numerator is the net displacement between positions from start to finish and the denominator is the length of the path traveled in that time period. The more often the target turns, the closer *tor* approaches 1. Group speed was calculated as the mean speed across all group members. Collective order, *ρ*, measures directional organization as the degree of alignment across all headings within the group:

$$\rho \left(t\right)=\frac{1}{25}\left|\sum_{i}{\hat{v}}_{i}\left(t\right)\right|,$$ (7)

where *ρ* at time *t* is given by the average magnitude of the orientation vectors for each prey in the group, ${\hat{v}}_{i}$. In the game the initial degree of directional organization is used as a factor, denoted by *ρ*_0_, whereas organization itself is denoted merely as *ρ*.

We again used linear mixed effects models in which variance within player was modeled as a random effect to account for our repeated measures design. When analyzing the relationship between our response variables (*P*_*L*_, *P*_*A*_) and the preys’ kinetic properties we were more interested in the relative contribution of these metrics to explaining any observed patterns than in their predictive abilities. As such, we standardized all kinetic metrics as $z-(\bar{z}/\sqrt{z}$) and scaled them to unit variance to compare their relative effects on player capture ability [48]. We checked for collinearity issues using both pairwise correlations and variable inflation factors (VIF)[49] and the only notable concern was a high correlation (Spearman *r* = 0.673) between target speed and group speed (See S3 Fig). To correct this we compared the fit of each of these metrics to *P*_*L*_ and *P*_*A*_ for each experimental session. We then used sequential regression to replace the values of the worse fitting predictor with the residuals obtained by regressing it onto the better predictor (determined using AIC)[50]. VIF values were then used to confirm the absence of any remaining collinearity in each session prior to model fitting.

## Results

### Session I: Target speed, social thresholds, and collective state

**Fig 2:**
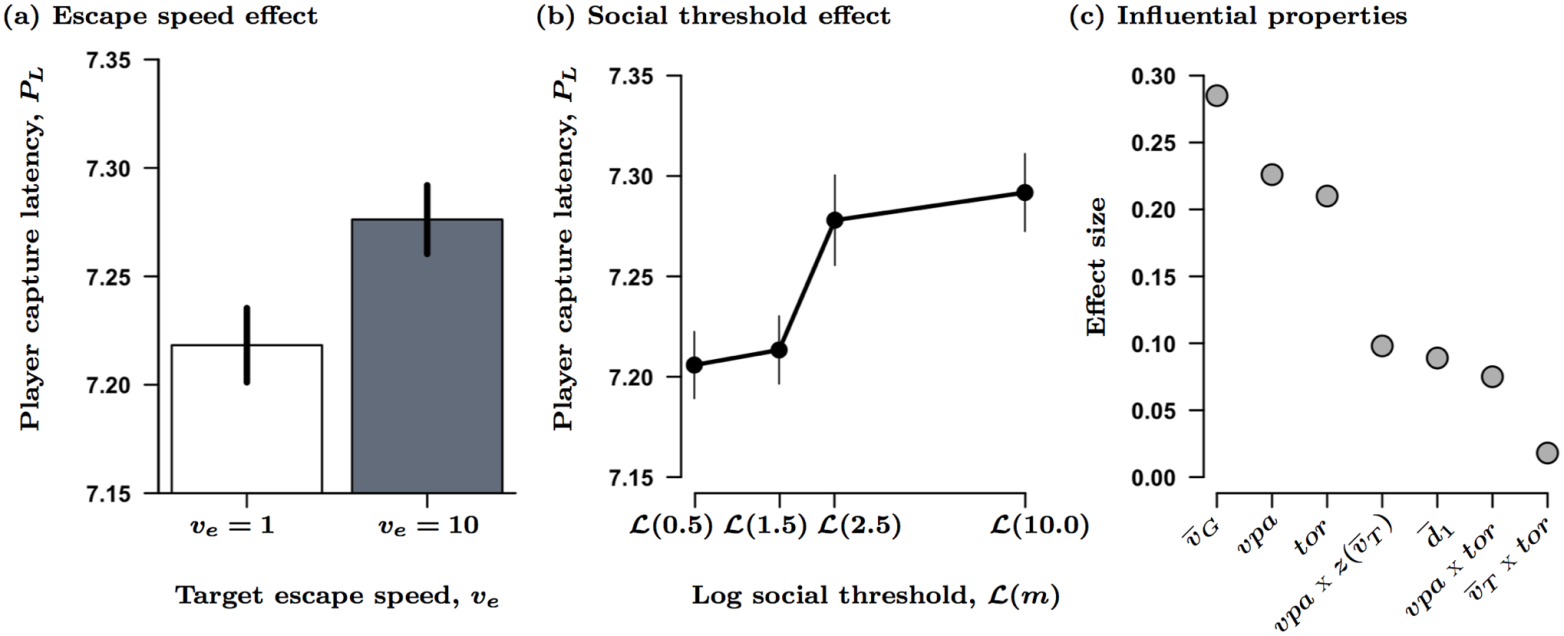
The effect of target escape speed (*v*_*e*_, a) and social interaction thresholds (*m*), b) on player capture latency, *P*_*L*_. Data in both (a) and (b) represent mean ± standard error (SE), corrected for repeated measures. In these trials prey groups were homogenous with members having the same social threshold (e.g., *m* = *m*_*T*_ = *m*_*G*_) and these values were transformed for the LMM analyses as L(m)=log(m+1). Figure (c) shows the relative effect size for each of the underlying individual and group level properties that significantly influenced *P*_*L*_. See Table 1 for a full list of all local and global variables and S2 Table for the LMM results for (c).

Players were slower to click on those targets adopting the faster of the two escape speeds (*P* = 0.003; Fig 2a; S2 Table). Player latency, *P*_*L*_, also increased with increases in the prey’s social threshold (*P* < 0.001; Fig 2b), yet the initial degree of organization in the prey had no significant effect on latency. The kinetic mechanisms driving the observed patterns in latency were primarily the overall speed of all prey (global effect), along with the spacing around the target and its turning behavior (local effect; Fig 2c). Taken together, these three kinetic properties accounted for over 73% of the overall effect strength in the LMM. Interestingly, while players took longer to capture targets in faster moving groups (*v*_*G*_, *P* < 0.001), the speed of the target itself had no effect. Players also took longer to click on targets that either turned more frequently (*tor*, *P* < 0.001) or were more spatially isolated (*vpa*, *P* < 0.001; ${\bar{d}}_{1}$, *P* < 0.001). Although local spacing significantly interacted with the targets’ speed and turning behavior, these interactions themselves had only a marginal impact on player capture latency (Fig 2c). Additionally, while increases in both local and global spacing had similar effects on player latency, changes in local spacing had a much stronger effect than did changes in global spacing (23% vs. 9% for *vpa* and ${\bar{d}}_{1}$, respectively; see S2 Table).

**Fig 3:**
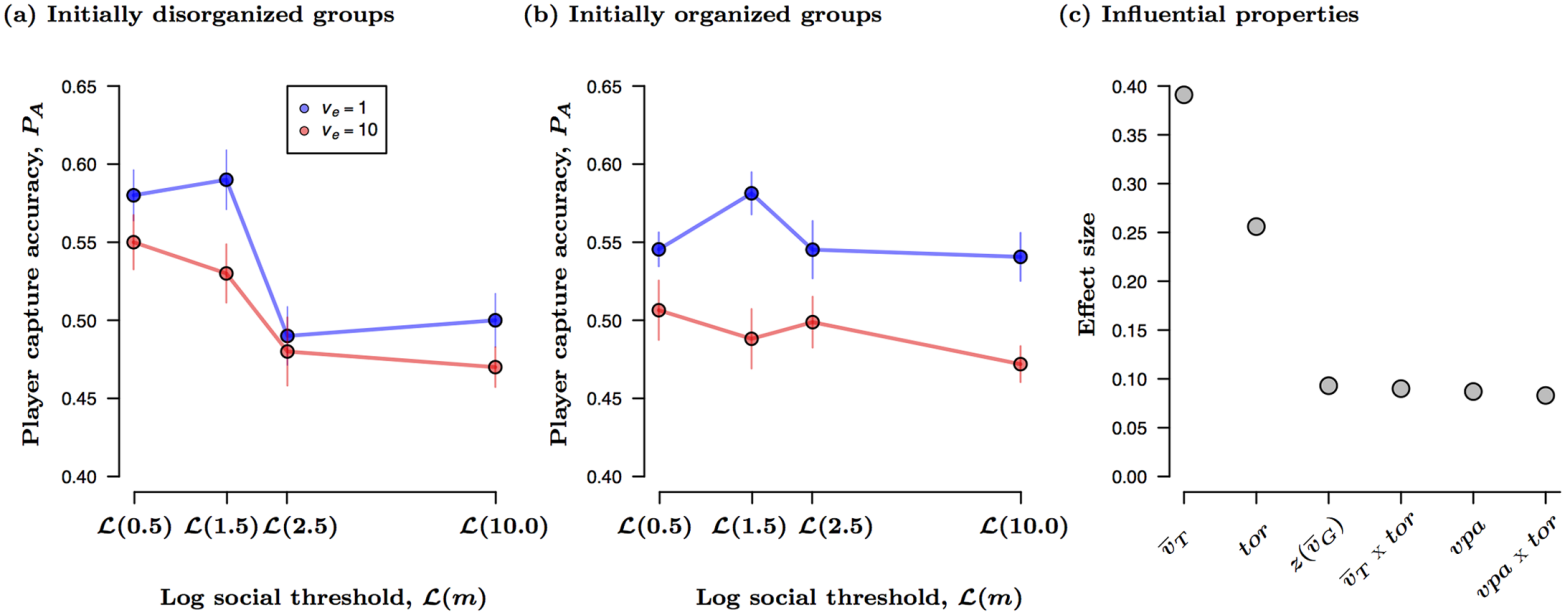
Effect of prey social threshold (*m*), target escape speed (*v*_*e*_), and the initial organization of the group (*ρ*_0_) on player capture accuracy, *P*_*A*_. All data points in (a) and (b) show mean ± SE, corrected for repeated measures. All prey share the same social threshold (e.g., *m* = *m*_*T*_ = *m*_*G*_) whose values were transformed as in Fig 2. Figure (c) shows the relative effect size for each of the underlying individual and group level properties that significantly influenced *P*_*A*_ (S3 Table).

Player accuracy, *P*_*A*_, improved when targets adopted slower escape speeds (*v*_*e*_, *P* < 0.001; Fig 3a, 3b; S3 Table), regardless of prey social threshold or the initial organizational state of the group. Accuracy decreased significantly as the prey’s social threshold increased and prey movements became less coordinated (*P* < 0.001). There was also a significant interaction between prey social thresholds and their initial overall degree of organization (*m* x *ρ*_0_, *P* = 0.002). Those virtual prey that were already organized (i.e., aligned) at the start of the attack were more difficult to catch than those that were disorganized at the start of the attack (*ρ*_0_, *P* < 0.01), and any socially derived effects on *P*_*A*_ dampened out relative to those observed when groups were initially disorganized (e.g., random initial orientations). In other words, while prey with higher social thresholds (less attention to group members) were always harder to capture than those with lower social thresholds (more attention to group members), the benefits of reduced social interactions were largely context dependent and were significantly reduced when groups were already organized (S4a Fig). In terms of the kinetic mechanisms driving these effects, target movements primarily drove player capture accuracy; the speed and turning behavior of the target accounted for 65% of the overall effect strength in the final LMM (Fig 3c; S3 Table).

### Session II: Sensory heterogeneity

Here we explored what happened when the target’s social threshold differed from that of the remaining group members, *m*_*T*_ ≠ *m*_*G*_, and we found that such sensory heterogeneity among the prey had no significant effect on player latency or accuracy. As in the trials of session I, players took longer to click on those targets that had higher social thresholds (*P* < 0.001, S4 Table). These prey paid less attention to their fellow group members, which resulted in less social coordination. Also, player accuracy was again affected by the interaction between the prey’s social threshold and the initial global organization of the group. While accuracy was lower for prey within groups that were initially organized (*P* < 0.001), and declined as the targets paid less attention to their group members (*P* < 0.001), these effects were much stronger for targets found in groups that were initially disorganized (*P* = 0.029; See S4b Fig and S5 Table). We found no substantive differences in the underlying kinetic metrics for either capture latency or accuracy, suggesting that the same movement behaviors driving the response variables in session I were doing the same here (S4 and S5 Tables).

### Session III: Visual confusion effect

**Fig 4:**
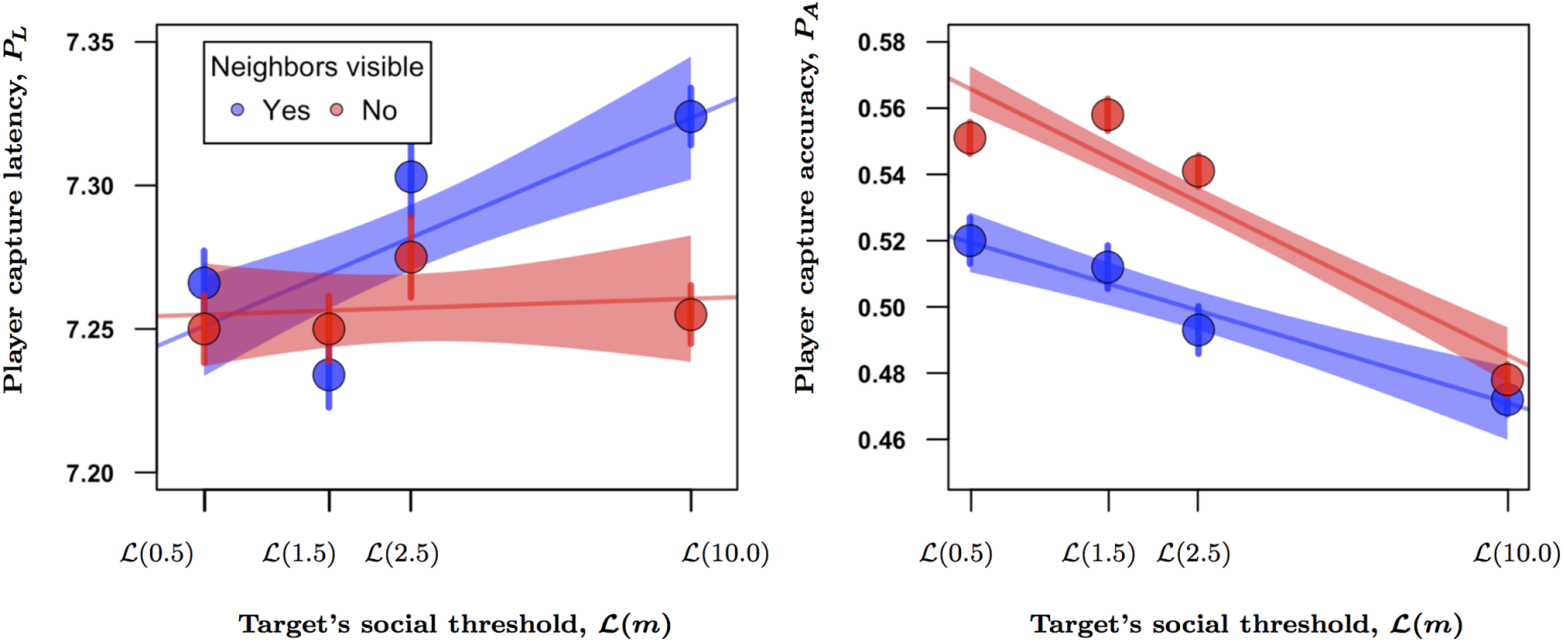
The confusion effect on player capture latency (a) and accuracy (b) as a function of target social threshold and the visibility of its neighbors. Trends are fit by regressing each response variable onto log(*m*_*T*_+1) with their 95% confidence intervals. Data points represent the means ± SE with error bars corrected for repeated measures.

Whether the targets appeared to be moving alone or within a group did not, by itself, have an overall effect on player latency. There was however a significant interaction between the targets’ social threshold and the veiled condition on player latency (*P* = 0.002). Although players were still slower to click on their targets as the prey’s social threshold increased (*P* < 0.001), this effect relied upon the visual presence of their neighbors and disappeared when the prey appeared to be traveling alone (Fig 4a; S6 Table). The behavioral kinetics driving player latency under these conditions were again mean group speed and target turning behavior (together making up 51% of the effect strength in the final LMM), and to a lesser degree, local spacing and individual speed (comprising another 32% of the effect strength).

In contrast to player latency, accuracy significantly increased simply because targets appeared to be moving alone (*P* < 0.001). While player accuracy retained its negative relationship with the prey’s social threshold, decreasing as *m* increased and prey showed less collective coordination, this effect was stronger for targets that appeared to be alone compared to those appearing in groups (*P* < 0.001; Fig 4b, S7 Table in the S1 Text). Player accuracy was again context dependent, improving when targets were found in groups that were initially disorganized rather than organized at the start of the attack (*P* < 0.001). The two most influential behavioral kinetics were target velocity (*P* < 0.001) and tortuosity (*P* < 0.001), which together accounted for 73% of the effect strength in the final LMM. The remaining influential kinetic factors included local spacing around the target (*vpa*, *P* < 0.001) and mean group velocity ($\bar{v_{G}}$, *P* < 0.001).

In general, the results of this last experiment indicate that an attacker’s visual confusion manifests at different stages of the attack sequence based on the degree of social coordination displayed by the prey—a finding that parallels previous scale-dependent effects of density on predator attacks [29]. Asocial motion, reminiscent of a flash expansion in fish, causes a visually driven delay in capture, but has little effect on the accuracy of the attack (asocial condition, *m* = 10, in Fig 4a *vs*. Fig 4b). In contrast, while any visible, coordinated motion around a targeted prey does little to delay an attack, it invariably leads to a visually driven reduction in capture accuracy, relative to when the targets were visually isolated (Fig 4, social conditions, *m* ≠ 10).

The results from this experiment also highlighted the visual component beneath a weak, but persistent, association between player latency and accuracy. In general, player accuracy got worse with time, as it was negatively correlated with capture latency (sessions I-III; Spearman *r* = −0.23, *P* < 0.001). The strength of this association varied across experimental sessions, but the trend remained consistent and weak. Here we found that any association between latency and accuracy disappeared when players were only able to see the targeted prey and not their surround neighbors (See S5 Fig).

## Discussion

The coordinated movements of social animals have long been seen as providing several adaptive advantages to group members, including enhanced prey awareness and increased predator response times [2–4]. Our results demonstrate for the first time that a visual sensory mechanism likely to enhance reflexive coordination in social animals can actually increase individual predation risk during a targeted attack. We also showed that changes in prey social coordination can visually confuse an attacker without requiring explicit changes in group size or density.

We controlled the social interactions in groups of virtual prey by tuning their visual sensory thresholds, which modulated their attention to neighbor activity and thereby influenced the degree of coordinated motion that emerged (Fig 1). We subsequently found a positive relationship between increases in coordinated, or polarized, motion and the individual’s risk of being captured (Figs 2b and 3a). A reduction in capture ability when grouped prey suddenly move with little to no regard to their neighbor’s decisions aligns well with expectations, given that such ‘flash expansions’ in fish schools are often associated with aiding in the confusion of a predator [14]. Yet the positive relationship between coordinated motion and an attacker’s capture ability deviates from general expectations. Coordinated collective motion is thought to benefit group members by enhancing the speed at which passive information is transmitted across individuals and has therefore been presumed to reduce predation risk [14, 51, 52]. Recent empirical evidence supports this to some degree. Ioannou and his colleagues showed that coordinated motion in virtual prey, meant to mimic water fleas, *Daphnia spp.*, reduced the likelihood of being attacked by Bluegill sunfish, *Lepomis macrochirus*[21]. However, this effect was primarily attributed to reducing the virtual preys’ exposure time to a hover-predator–a finding that is likely paralleled in our study by the interaction between capture ability and the initial degree of organization in the prey at the start of the attack (discussed further below). The findings of [21] also highlight a conflict across scales of organization, as those virtual prey in their study that showed more erratic turning behavior within a group were less prone to attack, yet at the group level the prevalence of such behaviors resulted in less mobile swarms that were preferentially attacked. In their model, social interaction influenced the virtual prey’s turning behavior, while speed was held constant.

**Fig 5:**
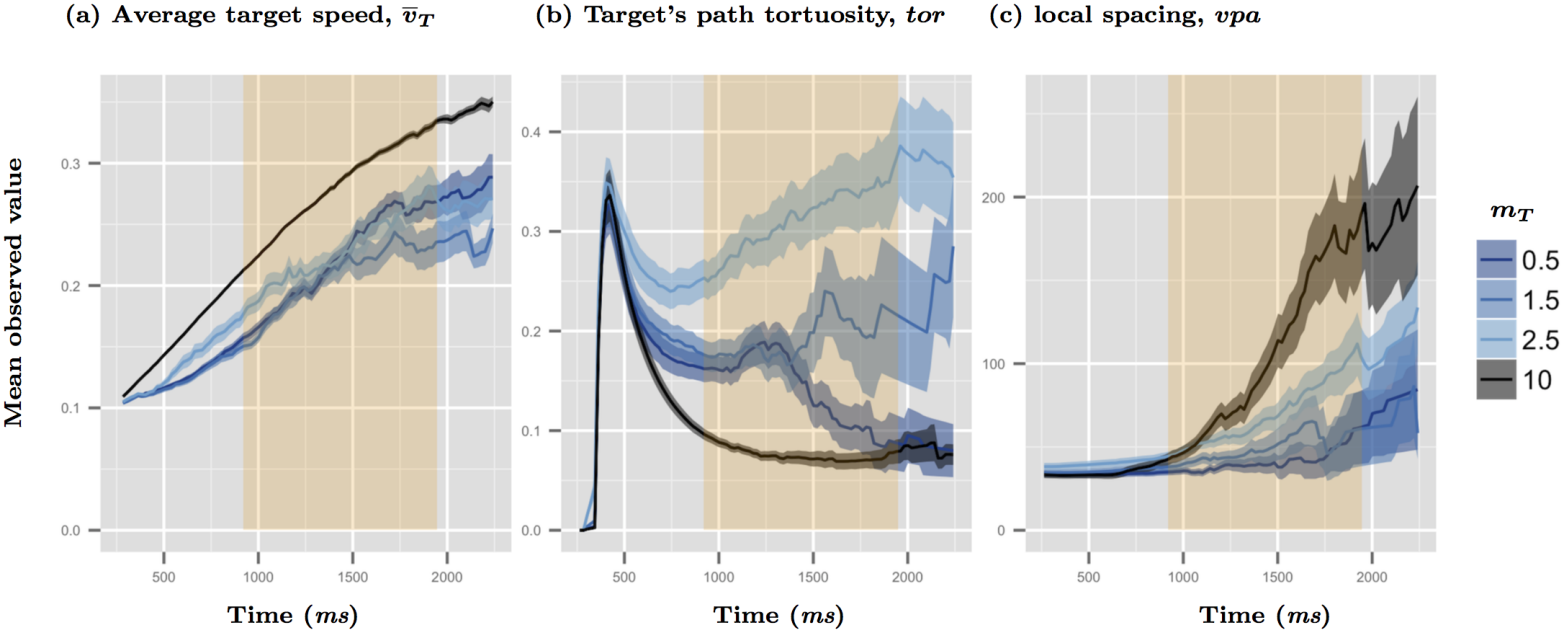
Time series of the kinetics underlying capture latency and accuracy. Data show temporal trends in average prey speed (a), path tortuosity (b), and local spacing around the targets (c). Data are pooled across initial organizational conditions (group organization, *ρ*_0_) and exclude escape sequences (e.g., *v*_*e*_ ≠ 10). Trend lines show the mean of each metric across replicates and their 95% confidence intervals. Vertical orange bands show the mean capture latency times for these conditions (mean ± 1 standard deviation, SD).

In our study reductions in player capture ability were generally attributed to increases in prey speed, while changes in prey turning behavior did not show a consistent relationship with capture ability. Less social prey initially moved more quickly than their more social counterparts, yet these same phenotypes also displayed very different turning behaviors from one another (see Fig 5a and 5b). Those prey with a modicum of social interaction displayed the most tortuous movements, while the asocial prey displayed the least (*m* = 2.5 *vs*. 10, respectively). In contrast, both of these sensory phenotypes resulted in consistently faster departure speeds in the window leading up to capture events, with asocial members moving the fastest. Speed also impacted player capture ability differently at different levels of organization. Capture latency was driven by the average speed of all group members, while accuracy was primarily affected by the target’s speed.

Players displayed behaviors that are typically consistent with confusion. They would occasionally track the wrong prey, or showed signs equivalent to the pass-along effect [5] in which the targeted prey’s movements lead an attacker to miss and accidentally hit one of the target’s neighbors (See S2d Fig). When our virtual prey appeared to be alone, socially mediated effects on capture latency were eliminated, despite the fact that the targets displayed identical movement patterns in the corresponding trials when their group members were visible to the player (Fig 4a). These results provide clear evidence that the loss of coordinated motion within a group of fixed size creates a visual confusion effect that impacts capture latency. More importantly, the data demonstrate that the degree of erratic motion in a targeted prey’s trajectory alone had no effect on how long it took players to capture their target. In contrast, changes in social movement patterns were sufficient to impact capture accuracy, although this effect was dampened by the presence of visual distractions (Fig 4b). So, as discussed briefly in session III of the results, we see that even though increases in social coordination enhance individual risk within groups, such behavior is none-the-less better than traveling alone. Additionally, in a drawn out engagement with a predator, maintaining some degree of social coordination may outweigh the benefits of the asocial condition because individuals would be less likely to become isolated from the group and preferentially targeted by either another predator, or a second attack.

In effect there are likely to be two opposing factors at play in predator confusion once a target has been selected from the group. The first is the loss of the target through misidentification (e.g., the pass along effect, S2d Fig). For example, [20] presented evidence that three-spined sticklebacks, *Gasterosteus aculeatus L*., were more prone to attack pairs of water fleas, *Daphnia magna*, when the prey moved in parallel compared to when their paths crossed. Another potential factor is that coordinated motion in a group of objects can enhance visual tracking. Consider that the simplest mechanism for visual tracking is to keep the target’s image steady on the retina [53], and this process is either reinforced by, or relies upon, visual saccades [54]. Visual saccades are rapid eye or head movements used to correct gaze errors, maintain fixation, or jump to predicted positions (insects, [18, 54]; crustaceans, [55]; fish, [56]; humans, [57]). Since target acquisition is temporarily lost during saccades, it is likely that target trajectories are more easily reacquired when any background stimuli move in a similar pattern [58]. Given our findings on what factors could lead to visual confusion in a predator, it would be informative for future efforts to adapt the current framework and explore how predators can compensate for these effects by switching targets and, if so, how this impacts prey behaviors or phenotypes.

As mentioned earlier, coordinated motion in our prey could reduce an attacker’s success since we found context dependent differences in the speed and accuracy of player capture ability. While targeting latency was robust to large changes in the initial state of the group (*ρ*_0_), capture accuracy was not and targets displaying escape speeds in polarized groups reduced player accuracy and likely drove the overall significant reduction in performance (Fig 3, S3 Table). Differences in targeting latency and accuracy patterns may be linked to the timing of events and the added importance that local spacing played in capture latency. Prey in our simulations self-organized quickly from an initially disorganized state (≈1 *s*; S6 Fig), and so these kinetic patterns occurred well within the time frame required by humans to both visually respond to stimuli (100–150 *ms*) and physically react through hand movements (≈300 *ms*; [59, 60]). Along with prey speed and tortuosity, local spacing was also an important factor driving capture latencies, and each of these three kinetic properties evolved differently over time (Fig 5). Differences in the speed and turning behavior of the prey emerged early during the attack sequence and diverged along the preys’ social thresholds, while the resulting impacts of these movements on local spacing (*vpa*) generally did not become apparent until after a time by which nearly half of the attacks were already over. Patterns in local spacing leading up to the capture window were also robust to differences in the initial conditions, while differences in speed and turning behavior were not (Fig 5, S6 Fig). In contrast, the resulting pattern in capture accuracy aligns well with our expectations, given the importance of collective speed and the relative reduction in capture accuracy across prey social thresholds (Figs 1b, 1c; 3a and 3b). When prey were already aligned at the start of an attack any socially mediated differences in target speeds were less apparent and differences in their respective tortuosities took longer to emerge (S6 Fig). Local spacing, it seems, plays a much more important role in the timing of an attack than in capture or handling.

Spacing has long been thought to play an important part in predation risk within social groups, particularly with regards to being more exposed than one’s neighbors and being singled out for attack [46]. The greatest potential risk in adopting a different movement strategy during an attack is becoming separated from the group, although prey on the outskirts can still benefit from the behavior of the group [41]. However, our results support the findings of [28], where increases in local prey density around a target served to increase the speed at which it was captured, rather than diminish it. In addition we found no evidence of a behavioral oddity effect, since there was no interaction between prey social thresholds within the groups (*m*_*T*_ x *m*_*G*_) and predator capture ability. Jones et al. [17] came to a similar conclusion in a study using human players to explore the effect of movement heterogeneity in asocial prey that were clumped together.

The approach presented here provides a means to control and test how sensory processes in both prey and predator can interact to influence their encounters. We have demonstrated a positive relationship between the degree of coordinated motion in social prey and the individual’s predation risk during a targeted attack. It is the degree of coordinated motion in a group of social prey that drives any visual delays in capture, while the accuracy of an attack relies primarily on the speed and turning actions of the targets themselves. Coordinated motion in animal groups can potentially reduce the time spent in dangerous areas and help individuals to avoid becoming isolated, yet such movement patterns can also alleviate predator confusion during a directed attack. The benefits of coordinated motion are therefore context dependent, which would help explain why social animals that move collectively display such a rich array of emergent behaviors during the course of an attack.

## Supporting Information

S1 Text Supplementary information containing additional details related to the model, game, analyses, and results. We review how visual perception and travel costs are incorporated into particle movement behaviors. The process of identifying and correcting edge effects in the game data is also reviewed and distinguished from perception driven events, such as confusion and the pass-along effect.
(PDF)

S1 Fig Generalized travel cost function. While travel cost (Equation s5 in the S1 Text) is symmetrical about v*, an organism’s optimal travel speed is closer to stationary than it is to its maximum potential, which results in more pronounced costs for exceeding v* (a). Fig. (b) shows how changes in travel costs are expected to vary linearly as a function of individual speed. Dashed lines represent the transition point as individuals shift between accelerating or decelerating, depending on their departure from v*. Fig. (c) shows a numerical simulation in which a single individual’s speed varies over time. The individual is initialized at sub-optimal travel speed, accelerates to its expected speed, then recovers from an imposed startle behavior. Parameters include: v* = 0.44, max{*v*} = 1.5 and *φ* = 0.1. Distances are scaled to body length, 2*r*, and time represents simulation steps. Additional parameters are found in S1 Table.
(PDF)

S2 Fig Movement patterns of both the simulated prey and player mouse activity in four different trials demonstrating instances of a trial error (a), edge effect (b), player confusion (c), and the pass-along effect (d). Grey circles represent the final positions of each virtual prey, with the target shown in orange. The mouse trajectory is shown in red, beginning with ‘x’ and ending with an open circle. We recorded only one instance of either subject or program error (a), where the player clearly tracks their target, but may simply not have pressed hard enough to trigger a click. In (b) the target manages to reach the safety of the boundary before the player could click on it (edge effect). The green triangle indicates the corrected point of capture, which is where the mouse was when the target crossed the boundary. (c) shows an example of the confusion effect where the player tracked the wrong particle. In (d) a near collision between the target and a neighbor causes them to separate from one another, thereby drastically altering the trajectories of these two prey. In this case the player initially drops down towards the target, but then switches to track and capture the neighbor (e.g., prey switching).
(PDF)

S3 Fig Pairwise Spearman rank correlations among all kinetic metrics (a) and the ranking of their absolute values (b). Pairwise correlation patterns varied very little across sessions, so we present the global patterns for generality (a). Only *v*_*T*_ and *v*_*G*_ showed any correlation of concern (*r* > 0.5 highlighted in red)[49]. A more conservative approach would also raise concern for a few moderate correlations (e.g, *r* ≥ 0.3, highlighted in yellow)[50]. However, once the *v*_*T*_ x *v*_*G*_ interaction was corrected using sequential regression none of the remaining metrics had VIF values greater than 2.6, indicating that there was no remaining collinearity [49].
(PDF)

S4 Fig The interactive effects of prey social thresholds and their initial organization on *P*_*A*_ in sessions I (a) and II (b). Prey groups in figure (a) were homogenous in their social thresholds (*m* = *m*_*T*_ = *m*_*G*_). Figure (b) shows the groups from session II, where the target’s social threshold in each trial differed from the remaining prey (*m*_*T*_ ≠ *m*_*G*_) and only the target’s social threshold had any significant effect on player capture ability (See S4 Table in the S1 Text).
(PDF)

S5 Fig Correlation between player accuracy and latency when targets appeared to be traveling in groups (a) or alone (b). Overall, player accuracy was only weakly correlated with capture latency. While the slope of this relationship varied across experimental conditions, the negative trend and its strength remained consistent. When targets appeared to be traveling alone (veiled condition, session III) the relationship was lost. Figure (a) shows the data from session III when the target’s neighbors were visible (Spearman *r* = −0.16, *S* = 21,453,141, *P* < 0.001), while (b) shows the pattern when the neighbors were hidden (Spearman *r* = 0.03, *S* = 17,796,466, *P* = 0.45).
(PDF)

S6 Fig Time series of the individual and group level kinetic metrics organized by their initial conditions. Additional conditions: *v*_*e*_ = 1.
(PDF)

S1 Video Example movie of the virtual swarms of prey displaying varying levels of coordinated motion. Groups are shown from an initially disorganized state for all social interaction threshold levels, *m*: {0.5, 1.5, 2.5, 10}. Additional model parameters are provided in S1 Table.
(MOV)

S1 Table Model parameters. (PDF)

S2 TableFinal linear mixed-models for player capture latency, *P*_*L*_, in session I for both the primary factors and kinetic metrics.Influential kinetic metrics in the final model are listed in order of effect size (SE—Standard Errors, DF—Degrees of Freedom). The notation $L(m_{\left\{T,G\right\}})$ is the natural log transformation of the adjusted threshold value *m*_*T*_ or *m*_*G*_ (e.g., log(*m*_{*T*,*G*}_ + 1)), while *z*() indicates that a metric was corrected for collinearity using sequential regression. For example, here *z*(*v*_*T*_) are the residuals from regressing *v*_*T*_ onto *v*_*G*_, which effectively allows us to retain any explanatory power that *v*_*T*_ contributes that isn’t already explained by *v*_*G*_.(PDF)

S3 Table Final linear mixed-models for player capture accuracy, *P*_*A*_, in session I. Notation and presentation are consistent with S2 Table.
(PDF)

S4 Table Final linear mixed-models for player capture latency, *P*_*L*_, in session II. Notation and presentation are consistent with S2 Table.
(PDF)

S5 Table Final linear mixed-models for player capture accuracy, *P*_*A*_, in session II. Notation and presentation are consistent with S2 Table.
(PDF)

S6 Table Final linear mixed-models for player capture latency, *P*_*L*_, in session III. Notation and presentation are consistent with S2 Table.
(PDF)

S7 Table Final linear mixed-models for player capture accuracy, *P*_*A*_, in session III. Notation and presentation are consistent with S2 Table.
(PDF)
