# Supplementary material for: A Sensory-Driven Trade-Off between Coordinated Motion in Social Prey and a Predator’s Visual Confusion
Source: PLoS Comput Biol. 2016 Feb 25;12(2):e1004708. doi: 10.1371/journal.pcbi.1004708 (PMC4767524; doi:10.1371/journal.pcbi.1004708)
Supplement: S1 Text — We review how visual perception and travel costs are incorporated into particle movement behaviors. The process of identifying and correcting edge effects in the game data is also reviewed and distinguished from perception driven events, such as confusion and the pass-along effect. (PDF) [file pcbi.1004708.s001.pdf]

Supplementary Information:
A sensory-driven tradeoff between coordinated motion in social prey and a predator's visual confusion

Lemasson, B.H[1*], Tanner, C.J. [2] and Dimperio, E. [3]

1. Environmental Laboratory, U.S. Army Engineer Research & Development Center, Santa Barbara, CA, USA[†]

2. Department of Ecology, Université de Lausanne, Switzerland[‡]

3. Corios, L.L.C., Portland, OR, U.S.A.

Contents

S1 Model 2
S1.1 Visual motion perception 2
S1.2 Travel costs 3
S2 Game 4
S2.1 Errors, edges, & ecologically relevant effects 4

*Author for correspondence: brilraven@gmail.com
†Current address: ERDC, Newport, OR, U.S.A.
‡Current address: School of Biological Sciences, University of Nebraska-Lincoln, NB, U.S.A.

S1 Model

S1.1 Visual motion perception

Here we review details of how visual perception is included in the model, which is based on earlier work [11] that we present here to help the reader. The speed at which objects appear to be moving is largely affected by how far away they are and in what direction they are moving. In a 2D world we assume that an object at distance $d_{j,i}$ will either be moving towards an observer, away from them, or across their line-of-sight. Line-of-sight in an unconstrained field of view is given by the directional vector $\mathbf{d}_{j,i}$, which is neighbor j's position with respect to observer i. The magnitude of this vector gives the intervening distance, $d_{j,i} = |\mathbf{d}_{j,i}|$, while the normalized component provides the direction, $\hat{\mathbf{d}}_{j,i} = \mathbf{d}_{j,i}/|\mathbf{d}_{j,i}|$. The observer thus sees a neighbor as an image on its retina whose motion has both a radial and translational component with regards to the observer's gaze:

$$\dot{\Omega}_{j,i} = f(\psi_{j,i}, \lambda_{j,i}) \tag{s1}$$

We assume that a neighbor's radial motion is given as a looming or receding stimuli:

$$\psi_{j,i} = \frac{-2 \cdot v^{\parallel}_{j,i} \cdot r}{d^2_{j,i} + r^2}, \tag{s2}$$

where $v^{\parallel}_{j,i}$ is the neighbor's speed along $\hat{\mathbf{d}}_{j,i}$ and r is the radius of the particles. Translational motion is given by:

$$\lambda_{j,i} = v^{\perp}_{j,i} \cdot d^{-1}_{j,i} \tag{s3}$$

where $v^{\perp}_{j,i}$ is the neighbor's speed across $\hat{\mathbf{d}}_{j,i}$ [11]. During each visual scan we assume that the overall strength of a neighbor's motion cue is proportional to the magnitude of these visual components at time step, t:

$$\omega_{j,i} = \sqrt{\psi^2_{j,i} + \lambda^2_{j,i}} \cdot \Delta t \tag{s4}$$

S1.2 Travel costs

The model builds upon a common assumption stemming from Brownian mechanics, namely that particles, and by extension organisms, vary their turning and speed behaviors independently [2]. Social models generally assume that individuals traveling in a group will turn to maintain a common direction with their neighbors, while adopting either a fixed or stochastic speed. Here we include a travel cost that forces individuals to dynamically adjust their speeds to reflect both energetic and ecological costs. The energetic, or metabolic, cost of travel is traditionally expressed as work and increases rapidly with incremental changes in speed across a wide range of taxa [3]. In addition, animals traveling in groups are more likely to be attacked when they fall behind and become separated from their neighbors – a risk that increases quickly with even small differences in distance [5]. These metabolic and ecological constraints can be combined into a generalized form by assuming a parabolic relationship between individual cost and travel speed:

$$\mathrm{c}(v) = \phi \left(\frac{v - v^*}{\max\{v\}} \right)^2 \tag{s5}$$

Travel cost changes when an individual's intended speed, v, deviates from their optimum, v^*. While any one individual's optimal travel speed may be unknown, it is reasonable to assume that this expected value is likely expressed by their average speed, $v^* = \overline{v}$. We also assume that individuals assess such changes relative to their own experienced limitations, given by $\max\{v\}$. As an individual's intended speed at time t is given by its socially influenced velocity, $\mathbf{v}_i^s$, v is defined as $v = |\mathbf{v}_i^s|$ from Eq. 4 of the main text[§]. Parameter ϕ is retained as the physical drag constant imposed by the surrounding media. Given the cost of travel (Figure S1a), it is likely that organisms will track how these costs vary in time and alter their behavior accordingly. We therefore assume that individuals will modify any socially motivated changes in speed in proportion to their relative changes in travel costs, γ:

$$\gamma = \frac{\delta \mathrm{c}}{\delta v} = 2\phi \left(\frac{v - v^*}{\max\{v\}^2} \right) \tag{s6}$$

The result is that γ changes linearly with speed as v deviates from v^*, which in turn causes individuals to either accelerate when $v < v^*$ or decelerate when $v > v^*$ (thereby

[§]Eq. 4 in the main text relates how individuals intend to respond to any socially motivated movements: $\mathbf{v}_i(t + \Delta t) = v_i^s \left[1 - \gamma(v_i^s)\right] \hat{\mathbf{v}}_i^s(t)$

imposing either a positive or negative feedback; Figure S1b, c).

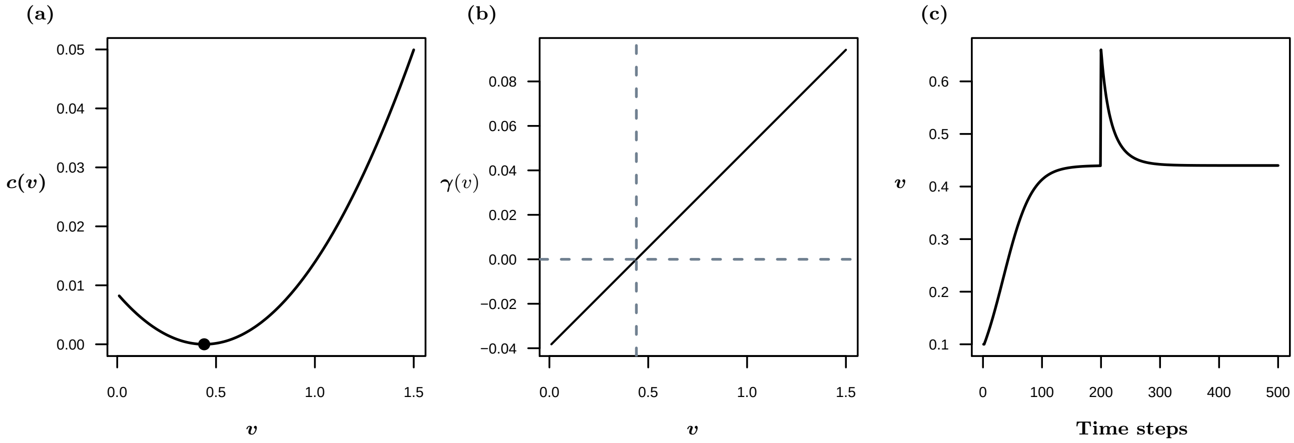

Figure S1: Generalized travel cost function. While travel cost (Equation (s5)) is symmetrical about v^*, an organism's optimal travel speed is closer to stationary than it is to it's maximum potential, which results in more pronounced costs for exceeding v^* (a). Fig. (b) shows how changes in travel costs are expected to vary linearly as a function of individual speed. Dashed lines represent the transition point as individuals shift between accelerating or decelerating, depending on their departure from v^*. Fig. (c) shows a numerical simulation in which a single individual's speed varies over time. The individual is initialized at sub-optimal travel speed, accelerates to its expected speed, then recovers from an imposed startle behavior. Parameters include: $v^* = 0.44$, $\max\{v\} = 1.5$ and $\phi = 0.1$. Distances are scaled to body length, $2r$, and time represents simulation steps. Additional parameters are found in Table S1

S2 Game

S2.1 Errors, edges, & ecologically relevant effects

In a limited number of cases the program failed to record a mouse-click during a trial, suggesting that the player did not attack their target. There were 13 such trials distributed across 11 players, with only two of these individuals repeating the behavior twice (out of 144 trials/player). Taken together these trials represented a very small subset of the data (13/4320 trials) and any spurious estimates of capture latency or accuracy were readily corrected. We visually reviewed the recorded movements of both the virtual prey and of the player's mouse in each of these cases. Only one trial appeared to stem from either player or program error, during which the player was clearly following the target, but stopped before the simulation ended (Figure S2a). This particular trial was excluded from our analyses. Player capture latency (P_L) and accuracy (P_A) in the remaining 12 trials were spurious due to edge effects and so were corrected and retained. In these scenarios a player would clearly follow a target, but fail to catch them before the target moved off-screen (e.g., Figure S2b). Edge effects also occurred when players moved their mouse back to the center of the screen before clicking, which was presumably an effort

to initiate the next trial (See Movie1 for an example of the protocol that instilled this behavior). These 'click' cases of edge effects were identified as outliers in the P_A data. In both click and non-click cases we recalculated both P_L and P_A based on where the mouse cursor was when the target escaped off-screen (green triangle in Figure S2b).

In addition to game-related errors, player tracking behavior also displayed characteristics indicative of either misidentification, or prey-switching. For example, in Figure S2c we see a player displaying signs of being confused by following the wrong particle. Players also occasionally would switch prey when a targeted particle came close to another, analogous to the so-called pass-along effect when a predator may switch targets during an attack (Figure S2d).

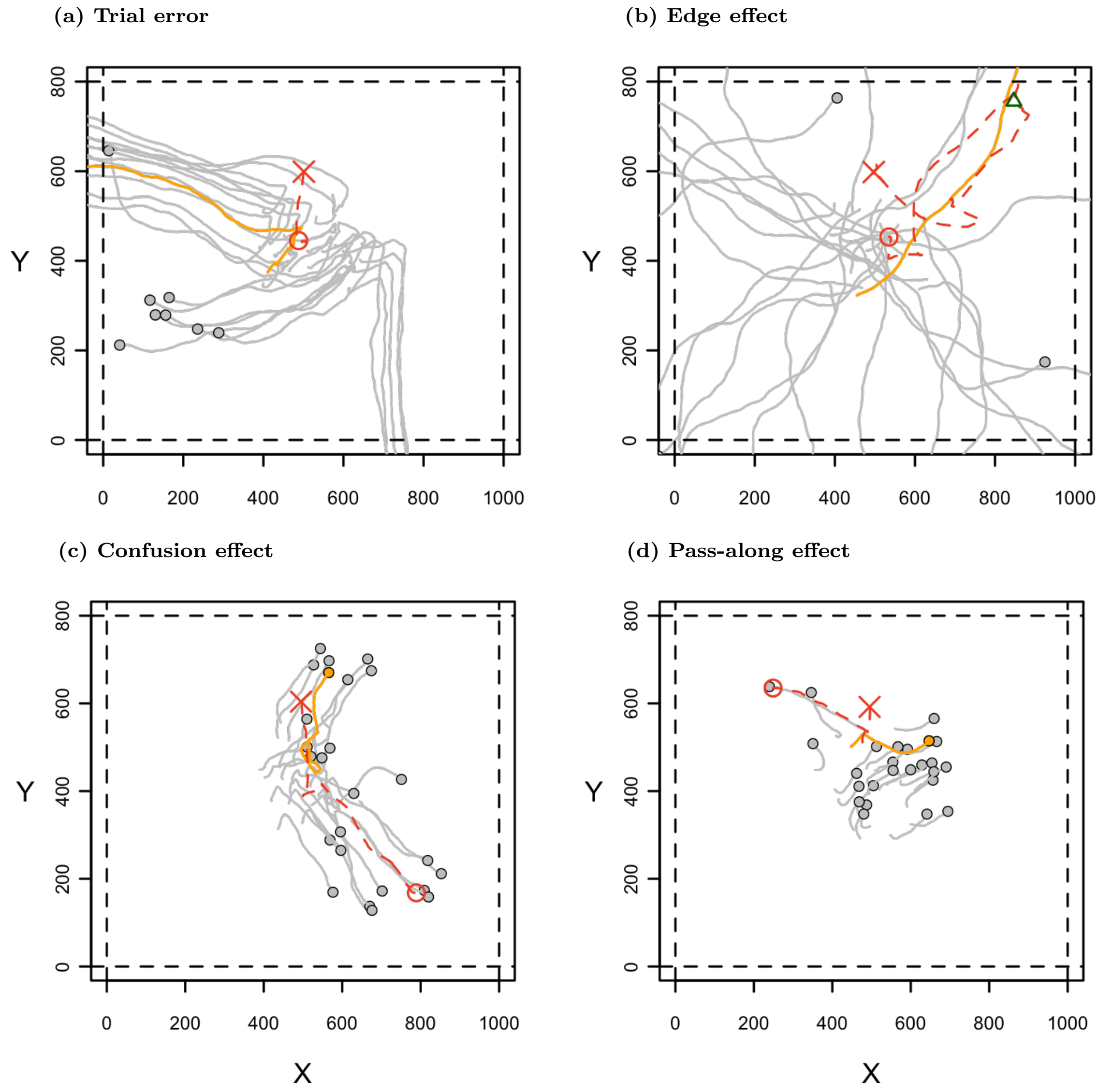

Figure S2: Movement patterns of both the simulated prey and player mouse activity in four different trials demonstrating instances of a trial error (a), edge effect (b), player confusion (c), and the pass-along effect (d). Grey circles represent the final positions of each virtual prey, with the target shown in orange. The mouse trajectory is shown in red, beginning with 'x' and ending with an open circle. We recorded only one instance of either subject or program error (a), where the player clearly tracks their target, but may simply not have pressed hard enough to trigger a click. In (b) the target manages to reach the safety of the boundary before the player could click on it (edge effect). The green triangle indicates the corrected point of capture which is where the mouse was when the target crossed the boundary. (c) shows an example of the confusion effect where the player tracked the wrong particle. In (d) a near collision between the target and a neighbor causes them to separate from one another, thereby drastically altering the trajectories of these two prey. In this case the player initially drops down towards the target, but then switches to track and capture the neighbor.

References

1. Lemasson B. H, Anderson J. J , Goodwin R. A (2013) Motion-guided attention promotes adaptive communications during social navigation. *Proc Roy Soc B* 280: 20122845.

2. Codling E, Plank M , Benhamou S (2008) Random walk models in biology. *J R Soc Interface* 5: 813–834.

3. Alexander R (1999) Bioenergetics: one price to run, swim or fly? *Nature* 397: 651–653.

4. Krause J, Ruxton G (2002) *Living in Groups* eds. Harvey P, May R (Oxford University Press, London, UK).

5. Romey W, Walston A , Watt P (2008) Do 3-D predators attack the margins of 2-D selfish herds? *Behav Ecol* 19: 74–78.
