# Supplementary figures and images for: A Sensory-Driven Trade-Off between Coordinated Motion in Social Prey and a Predator’s Visual Confusion
Source: PLoS Comput Biol. 2016 Feb 25;12(2):e1004708. doi: 10.1371/journal.pcbi.1004708 (PMC4767524; doi:10.1371/journal.pcbi.1004708)

(a)

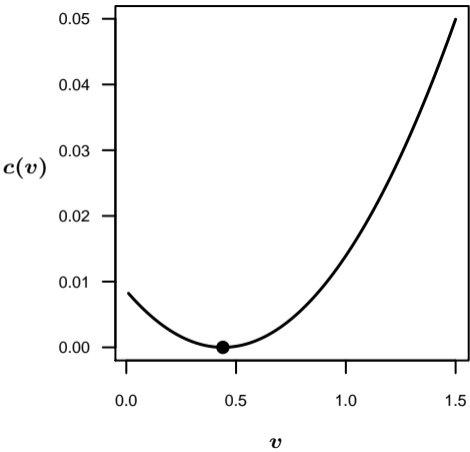

(b)

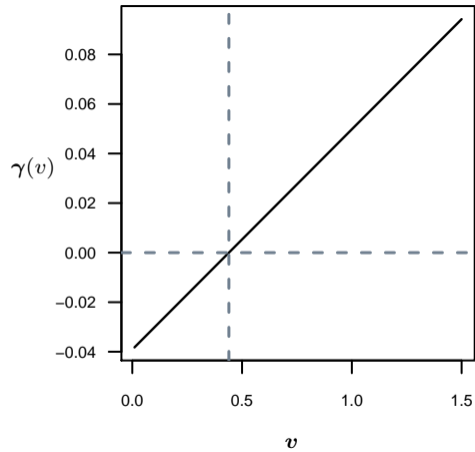

(c)

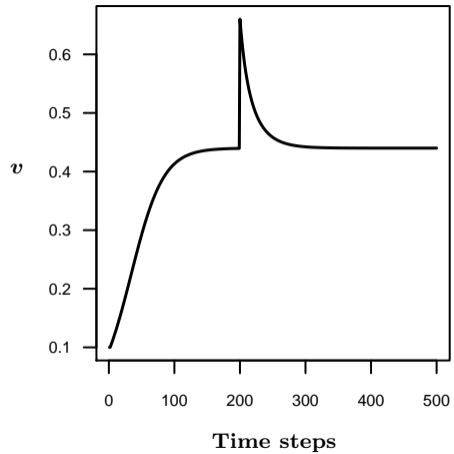

Supplement: S1 Fig — While travel cost (Equation s5 in the S1 Text) is symmetrical about v*, an organism’s optimal travel speed is closer to stationary than it is to its maximum potential, which results in more pronounced costs for exceeding v* (a). Fig. (b) shows how changes in travel costs are expected to vary linearly as a function of individual speed. Dashed lines represent the transition point as individuals shift between accelerating or decelerating, depending on their departure from v*. Fig. (c) shows a numerical simulation in which a single individual’s speed varies over time. The individual is initialized at sub-optimal travel speed, accelerates to its expected speed, then recovers from an imposed startle behavior. Parameters include: v* = 0.44, max{v} = 1.5 and φ = 0.1. Distances are scaled to body length, 2r, and time represents simulation steps. Additional parameters are found in S1 Table. (PDF) [file pcbi.1004708.s002.pdf]

(a) Trial error

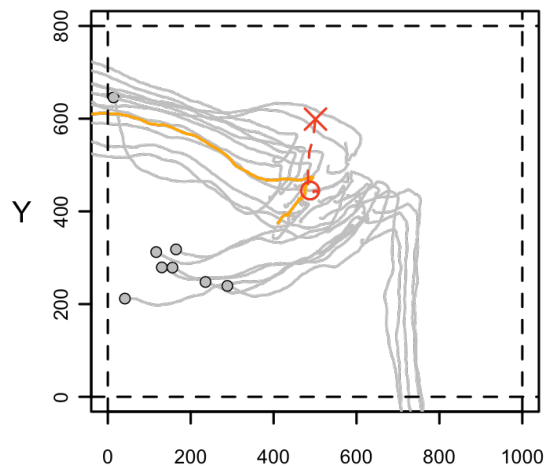

(b) Edge effect

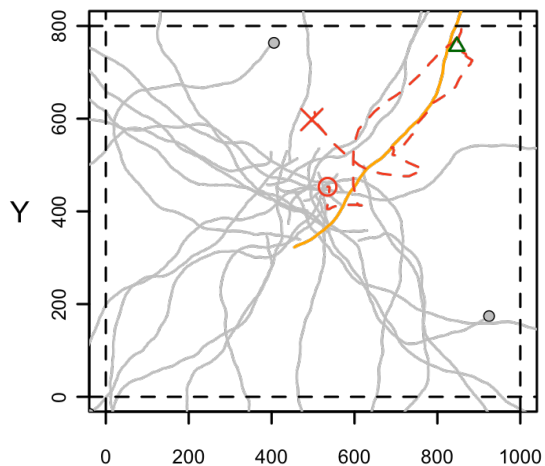

(c) Confusion effect

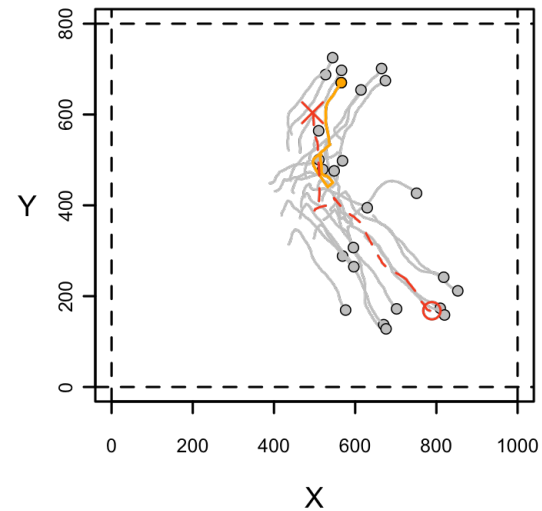

(d) Pass-along effect

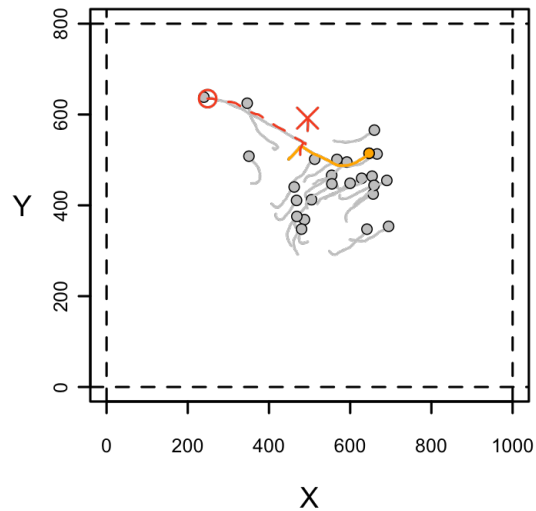

Supplement: S2 Fig — Grey circles represent the final positions of each virtual prey, with the target shown in orange. The mouse trajectory is shown in red, beginning with ‘x’ and ending with an open circle. We recorded only one instance of either subject or program error (a), where the player clearly tracks their target, but may simply not have pressed hard enough to trigger a click. In (b) the target manages to reach the safety of the boundary before the player could click on it (edge effect). The green triangle indicates the corrected point of capture, which is where the mouse was when the target crossed the boundary. (c) shows an example of the confusion effect where the player tracked the wrong particle. In (d) a near collision between the target and a neighbor causes them to separate from one another, thereby drastically altering the trajectories of these two prey. In this case the player initially drops down towards the target, but then switches to track and capture the neighbor (e.g., prey switching). (PDF) [file pcbi.1004708.s003.pdf]

(a)

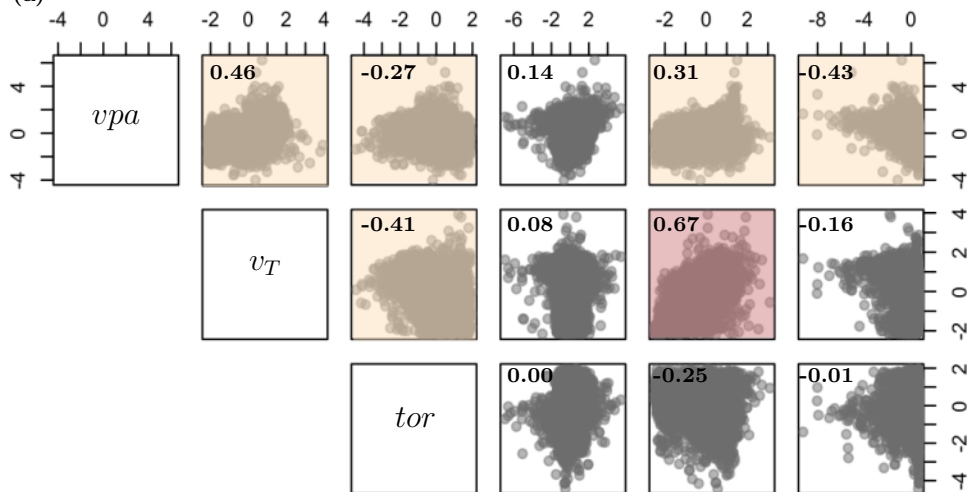

(b)

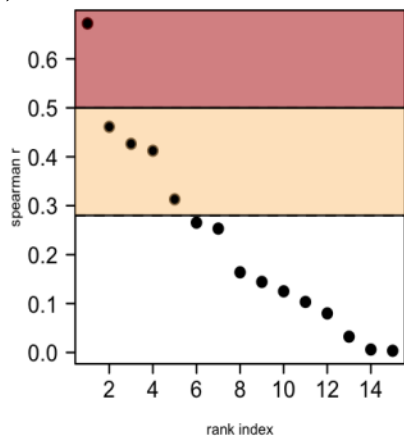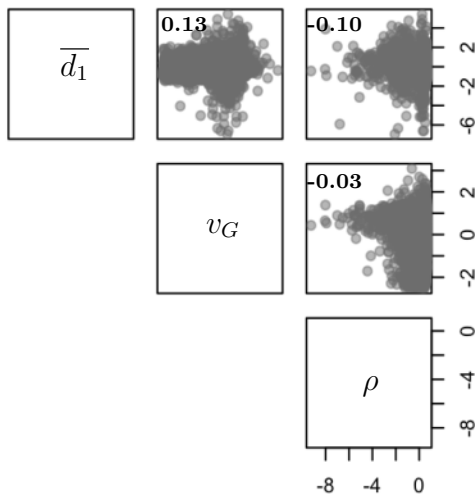

Supplement: S3 Fig — Pairwise correlation patterns varied very little across sessions, so we present the global patterns for generality (a). Only vT and vG showed any correlation of concern (r > 0.5 highlighted in red)[49]. A more conservative approach would also raise concern for a few moderate correlations (e.g, r ≥ 0.3, highlighted in yellow)[50]. However, once the vT x vG interaction was corrected using sequential regression none of the remaining metrics had VIF values greater than 2.6, indicating that there was no remaining collinearity [49]. (PDF) [file pcbi.1004708.s004.pdf]

(a)

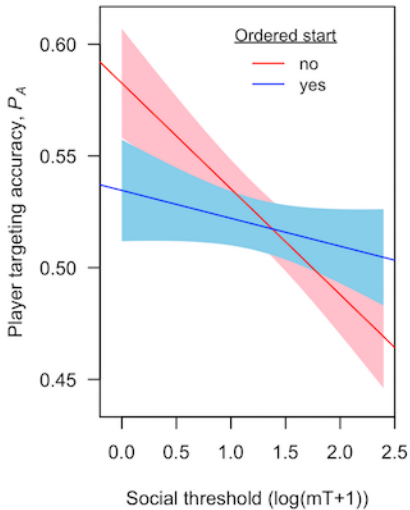

(b)

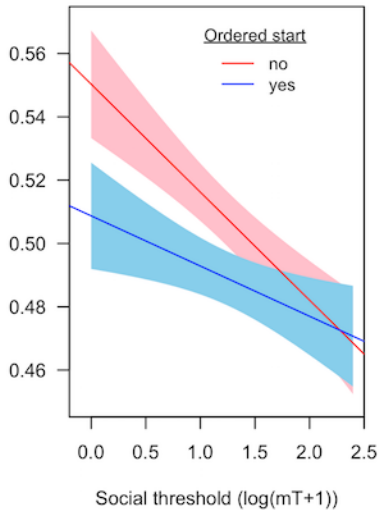

Supplement: S4 Fig — Prey groups in figure (a) were homogenous in their social thresholds (m = mT = mG). Figure (b) shows the groups from session II, where the target’s social threshold in each trial differed from the remaining prey (mT ≠ mG) and only the target’s social threshold had any significant effect on player capture ability (See S4 Table in the S1 Text). (PDF) [file pcbi.1004708.s005.pdf]

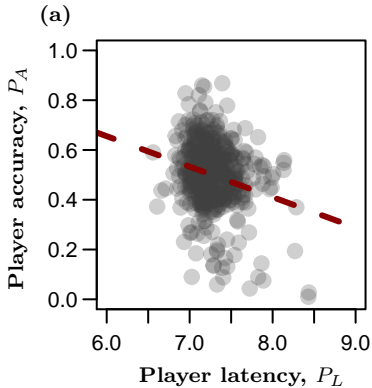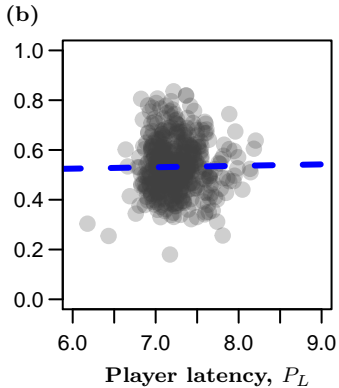

Supplement: S5 Fig — Overall, player accuracy was only weakly correlated with capture latency. While the slope of this relationship varied across experimental conditions, the negative trend and its strength remained consistent. When targets appeared to be traveling alone (veiled condition, session III) the relationship was lost. Figure (a) shows the data from session III when the target’s neighbors were visible (Spearman r = −0.16, S = 21,453,141, P < 0.001), while (b) shows the pattern when the neighbors were hidden (Spearman r = 0.03, S = 17,796,466, P = 0.45). (PDF) [file pcbi.1004708.s006.pdf]

Groups initially disorganized

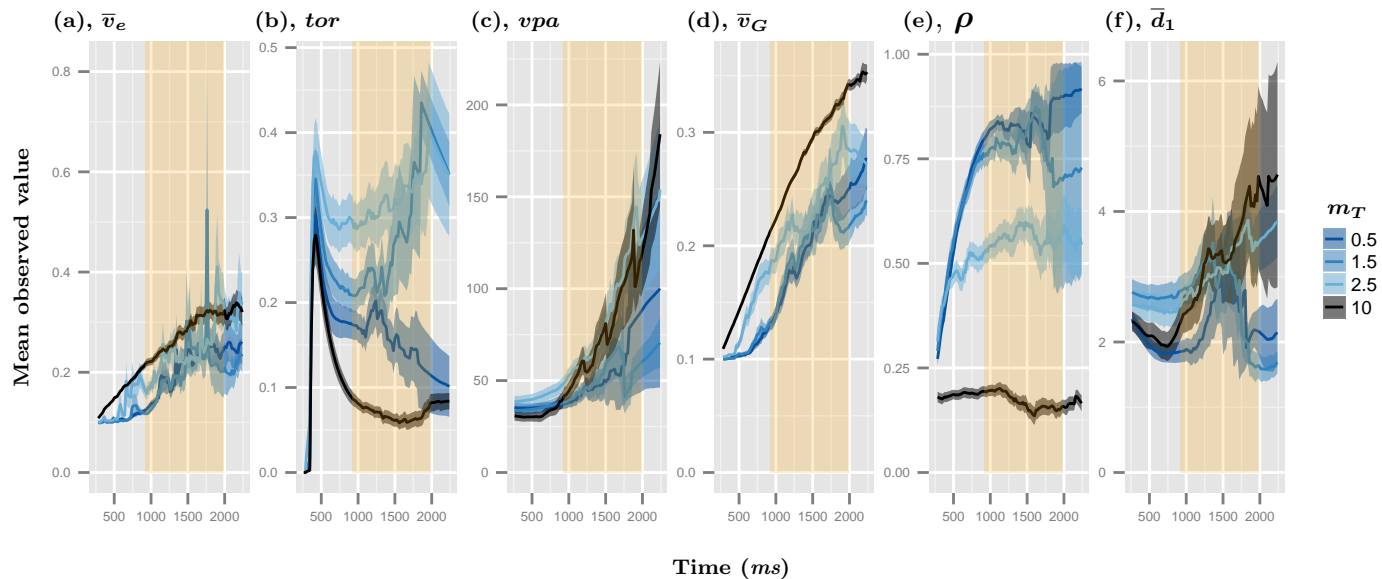

Groups initially organized

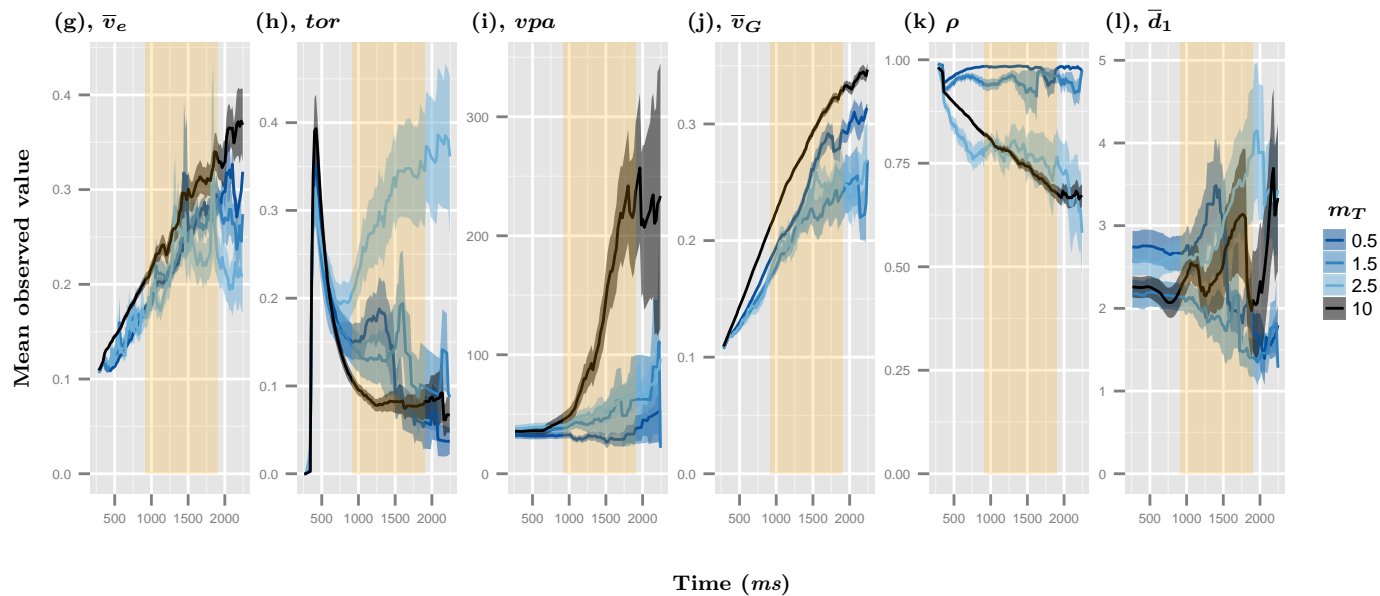

Supplement: S6 Fig — Additional conditions: ve = 1. (PDF) [file pcbi.1004708.s007.pdf]
