# Supplementary material for: A Sensory-Driven Trade-Off between Coordinated Motion in Social Prey and a Predator’s Visual Confusion
Source: PLoS Comput Biol. 2016 Feb 25;12(2):e1004708. doi: 10.1371/journal.pcbi.1004708 (PMC4767524; doi:10.1371/journal.pcbi.1004708)
Supplement: S1 Table — (PDF) [file pcbi.1004708.s009.pdf]

Notation	Description	Values	Units
G	Group size	25	-
r	Particle radius	0.5	D
Ω_{min}	Minimum detectable image size	0.009	radians
m	Motion threshold	[0.0, 1.5, 2.5, 10]	-
α	Maximum turning arc	$\pm\ \pi/2$	radians
t	Time step	1	-
η	Noise strength	0.1	-
v	Min/mean/max travel speed	[0.1, 0.4, 1.5]	$D \cdot t^{-1}$
ϕ	Drag coefficient	0.1	-
