# Supplementary material for: A Sensory-Driven Trade-Off between Coordinated Motion in Social Prey and a Predator’s Visual Confusion
Source: PLoS Comput Biol. 2016 Feb 25;12(2):e1004708. doi: 10.1371/journal.pcbi.1004708 (PMC4767524; doi:10.1371/journal.pcbi.1004708)
Supplement: S2 Table — Influential kinetic metrics in the final model are listed in order of effect size (SE—Standard Errors, DF—Degrees of Freedom). The notation L(m{T,G}) is the natural log transformation of the adjusted threshold value mT or mG (e.g., log(m{T,G} + 1)), while z() indicates that a metric was corrected for collinearity using sequential regression. For example, here z(vT) are the residuals from regressing vT onto vG, which effectively allows us to retain any explanatory power that vT contributes that isn’t already explained by vG. (PDF) [file pcbi.1004708.s010.pdf]

Primary factors

	Value	SE	DF	t-value	p-value
(Intercept)	7.162	0.026	928	270.917	< 0.001
v_e	0.058	0.017	928	3.488	0.001
$\mathcal{L}(m_T)$	0.046	0.011	928	4.009	< 0.001

Kinetic metrics

	Value	SE	DF	t-value	p-value	Effect size
(Intercept)	7.277	0.017	923	421.470	< 0.001	–
v_G	0.086	0.008	923	11.050	< 0.001	0.285
vpa	0.069	0.008	923	8.412	< 0.001	0.226
tor	0.064	0.009	923	6.973	< 0.001	0.210
vpa x $z(v_T)$	-0.030	0.010	923	-2.845	0.005	0.098
$\overline{d}_1$	0.027	0.008	923	3.420	0.001	0.089
vpa x tor	0.023	0.009	923	2.574	0.010	0.075
