# Supplementary material for: A Sensory-Driven Trade-Off between Coordinated Motion in Social Prey and a Predator’s Visual Confusion
Source: PLoS Comput Biol. 2016 Feb 25;12(2):e1004708. doi: 10.1371/journal.pcbi.1004708 (PMC4767524; doi:10.1371/journal.pcbi.1004708)
Supplement: S3 Table — Notation and presentation are consistent with S2 Table. (PDF) [file pcbi.1004708.s011.pdf]

Primary factors

	Value	SE	DF	t-value	p-value
(Intercept)	0.602	0.013	926	46.556	< 0.001
v_e	-0.048	0.008	926	-5.888	< 0.001
ρ_0	-0.041	0.016	926	-2.545	0.011
$\mathcal{L}(m_T)$	-0.041	0.016	926	-2.545	0.011
ρ_0 x $\mathcal{L}(m_T)$	0.032	0.011	926	2.835	0.005

Kinetic metrics

	Value	SE	DF	t-value	p-value	Effect size
(Intercept)	0.513	0.006	924	79.791	< 0.001	–
v_T	-0.052	0.005	924	-11.479	< 0.001	0.391
tor	-0.034	0.005	924	-7.273	< 0.001	0.256
$z(v_G)$	-0.012	0.006	924	-2.244	0.025	0.093
v_T x tor	0.012	0.005	924	2.636	0.009	0.090
vpa	-0.012	0.004	924	-2.723	0.007	0.087
vpa x tor	-0.011	0.005	924	-2.346	0.019	0.083
