# Supplementary material for: A Sensory-Driven Trade-Off between Coordinated Motion in Social Prey and a Predator’s Visual Confusion
Source: PLoS Comput Biol. 2016 Feb 25;12(2):e1004708. doi: 10.1371/journal.pcbi.1004708 (PMC4767524; doi:10.1371/journal.pcbi.1004708)
Supplement: S4 Table — Notation and presentation are consistent with S2 Table. (PDF) [file pcbi.1004708.s012.pdf]

Primary factors

	Value	SE	DF	t-value	p-value
(Intercept)	7.236	0.020	1889	356.825	< 0.001
$\mathcal{L}(m_T)$	0.036	0.007	1889	4.881	< 0.001

Kinetic metrics

	Value	SE	DF	t-value	p-value	Effect Size
(Intercept)	7.279	0.015	1884	486.896	< 0.001	–
v_G	0.073	0.005	1884	13.483	< 0.001	0.271
tor	0.064	0.005	1884	11.857	< 0.001	0.239
vpa	0.055	0.006	1884	9.955	< 0.001	0.203
$z(v_T)$	-0.039	0.008	1884	-4.968	< 0.001	0.144
$z(v_T)$ x tor	0.021	0.007	1884	2.951	0.003	0.079
$\overline{d_1}$	0.017	0.005	1884	3.474	0.001	0.064
