# Supplementary material for: A Sensory-Driven Trade-Off between Coordinated Motion in Social Prey and a Predator’s Visual Confusion
Source: PLoS Comput Biol. 2016 Feb 25;12(2):e1004708. doi: 10.1371/journal.pcbi.1004708 (PMC4767524; doi:10.1371/journal.pcbi.1004708)
Supplement: S5 Table — Notation and presentation are consistent with S2 Table. (PDF) [file pcbi.1004708.s013.pdf]

Primary factors

	Value	SE	DF	t-value	p-value
(Intercept)	0.550	0.009	1887	61.402	< 0.001
$\mathcal{L}(m_T)$	-0.034	0.006	1887	-5.826	< 0.001
ρ_0	-0.041	0.012	1887	-3.453	0.001
$\mathcal{L}(m_T)$ x ρ_0	0.018	0.008	1887	2.255	0.024

Kinetic metrics

	Value	SE	DF	t-value	p-value	Effect Size
(Intercept)	0.504	0.005	1884	104.706	< 0.001	–
v_T	-0.048	0.003	1884	-13.867	< 0.001	0.369
tor	-0.037	0.003	1884	-11.639	< 0.001	0.283
v_T x tor	0.013	0.003	1884	3.767	< 0.001	0.096
vpa x tor	-0.012	0.003	1884	-3.741	< 0.001	0.095
tor	-0.012	0.003	1884	-3.977	< 0.001	0.093
$z(v_G)$	-0.008	0.004	1884	-1.990	0.047	0.063
