# Supplementary material for: A Sensory-Driven Trade-Off between Coordinated Motion in Social Prey and a Predator’s Visual Confusion
Source: PLoS Comput Biol. 2016 Feb 25;12(2):e1004708. doi: 10.1371/journal.pcbi.1004708 (PMC4767524; doi:10.1371/journal.pcbi.1004708)
Supplement: S6 Table — Notation and presentation are consistent with S2 Table. (PDF) [file pcbi.1004708.s014.pdf]

Primary factors

	Value	SE	DF	t-value	p-value
(Intercept)	7.236	0.020	3806	355.321	< 0.001
$\mathcal{L}(m_T)$	0.036	0.008	3806	4.804	< 0.001
$\mathcal{L}(m_T)$ x veiled	-0.033	0.011	3806	-3.125	0.002

Kinetic metrics

	Value	SE	DF	t-value	p-value	Effect Size
(Intercept)	7.272	0.014	3801.000	508.583	< 0.001	–
v_G	0.077	0.004	3801.000	20.109	< 0.001	0.256
tor	0.075	0.004	3801.000	19.873	< 0.001	0.251
vpa	0.059	0.004	3801.000	14.415	< 0.001	0.195
$z(v_T)$	-0.038	0.006	3801.000	-6.911	< 0.001	0.127
$z(v_T)$ x tor	0.016	0.005	3801.000	3.147	0.002	0.054
$\overline{d_1}$	0.014	0.003	3801.000	4.136	< 0.001	0.048
vpa x $z(v_T)$	-0.013	0.006	3801.000	-2.234	0.026	0.043
ρ	0.008	0.004	3801.000	2.130	0.033	0.026
