# Supplementary material for: A Sensory-Driven Trade-Off between Coordinated Motion in Social Prey and a Predator’s Visual Confusion
Source: PLoS Comput Biol. 2016 Feb 25;12(2):e1004708. doi: 10.1371/journal.pcbi.1004708 (PMC4767524; doi:10.1371/journal.pcbi.1004708)
Supplement: S7 Table — Notation and presentation are consistent with S2 Table. (PDF) [file pcbi.1004708.s015.pdf]

Primary factors

	Value	SE	DF	t-value	p-value
(Intercept)	0.548	0.007	3804.000	76.521	< 0.001
$\mathcal{L}(m_T)$	-0.034	0.004	3804.000	-7.758	< 0.001
veiled	0.053	0.007	3804.000	7.204	< 0.001
ρ_0	-0.037	0.007	3804.000	-5.064	< 0.001
$\mathcal{L}(m_T)$ x veiled	-0.016	0.005	3804.000	-3.142	0.002
$\mathcal{L}(m_T)$ x ρ_0	0.020	0.005	3804.000	3.850	< 0.001

Kinetic metrics

	Value	SE	DF	t-value	p-value	Effect Size
(Intercept)	0.522	0.004	3804	130.137	< 0.001	–
v_T	-0.048	0.002	3804	-21.856	< 0.001	0.501
tor	-0.022	0.002	3804	-11.001	< 0.001	0.228
v_T x tor	0.010	0.002	3804	4.740	< 0.001	0.100
$z(v_G)$	-0.009	0.003	3804	-3.533	< 0.001	0.095
vpa	-0.007	0.002	3804	-3.697	< 0.001	0.077
